# Claudin-2 protects against colitis-associated cancer by promoting colitis-associated mucosal healing

**DOI:** 10.1172/JCI170771

**Published:** 2023-12-01

**Authors:** Rizwan Ahmad, Balawant Kumar, Ishwor Thapa, Raju Lama Tamang, Santosh K. Yadav, Mary K. Washington, Geoffrey A. Talmon, Alan S. Yu, Dhundy K. Bastola, Punita Dhawan, Amar B. Singh

**Affiliations:** 1 Department of Biochemistry and Molecular Biology, University of Nebraska Medical Center, Omaha, Nebraska, USA.; 2 School of Interdisciplinary Informatics, University of Nebraska Omaha, Omaha, Nebraska, USA.; 3 Department of Pathology, Microbiology and Immunology, Vanderbilt University Medical Center, Nashville, Tennessee, USA.; 4 Department of Pathology and Microbiology, University of Nebraska Medical Center, Omaha, Nebraska, USA.; 5 Jared Grantham Kidney Institute, University of Kansas Medical Center, Kansas City, Kansas, USA.; 6 Fred and Pamela Buffett Cancer Center, University of Nebraska Medical Center, Omaha, Nebraska, USA.; 7 VA Nebraska-Western Iowa Health Care System, Omaha, Nebraska, USA.

**Keywords:** Gastroenterology, Colorectal cancer, Inflammatory bowel disease, Tight junctions

## Abstract

Patients with inflammatory bowel disease (IBD) are susceptible to colitis-associated cancer (CAC). Chronic inflammation promotes the risk for CAC. In contrast, mucosal healing predicts improved prognosis in IBD and reduced risk of CAC. However, the molecular integration among colitis, mucosal healing, and CAC remains poorly understood. Claudin-2 (CLDN2) expression is upregulated in IBD; however, its role in CAC is not known. The current study was undertaken to examine the role for CLDN2 in CAC. The AOM/DSS-induced CAC model was used with WT and CLDN2-modified mice. High-throughput expression analyses, murine models of colitis/recovery, chronic colitis, ex vivo crypt culture, and pharmacological manipulations were employed in order to increase our mechanistic understanding. The *Cldn2*KO mice showed significant inhibition of CAC despite severe colitis compared with WT littermates. *Cldn2* loss also resulted in impaired recovery from colitis and increased injury when mice were subjected to intestinal injury by other methods. Mechanistic studies demonstrated a possibly novel role of CLDN2 in promotion of mucosal healing downstream of EGFR signaling and by regulation of Survivin expression. An upregulated CLDN2 expression protected from CAC and associated positively with crypt regeneration and Survivin expression in patients with IBD. We demonstrate a potentially novel role of CLDN2 in promotion of mucosal healing in patients with IBD and thus regulation of vulnerability to colitis severity and CAC, which can be exploited for improved clinical management.

## Graphical abstract

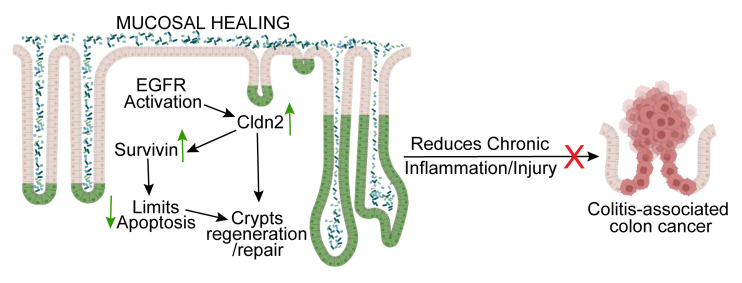

## Introduction

Patients with inflammatory bowel disease (IBD) have two big fears: (a) developing colon cancer and (b) losing their colon by colectomy because of the heightened risk of colitis-associated cancer (CAC) (1, 2). CAC is driven by the continuous exposure to intestinal inflammation (3, 4). Thus, mechanism(s) that can prevent recurrent inflammation in IBD can also protect against CAC. Mucosal healing (MH), a critical endpoint in the clinical management of IBD, is associated with improved prognosis, including reduced risk of CAC (5). Epithelial cell survival, proliferation, and migration play integrated roles during MH and functional crypt formation. This phase is promoted by growth factors, including EGF and keratinocyte growth factor (KGF), and immune cytokines, such as IL-22 (6). However, details regarding the molecular regulation of MH and how it alters the outcomes for CAC remain poorly understood.

Claudin-2 (CLDN2) expression is upregulated in IBD (7–9). However, modeling of the pathological CLDN2 expression, as in IBD, protects mice from dextran sulfate sodium–induced (DSS-induced) or C. rodentium–induced colitis (10, 11). The KO of endogenous CLDN2 expression promotes colitis (11, 12). Interestingly, when subjected to the T cell colitis, the offspring of Villin-*Cldn2*TG and *Rag1*KO mice show severe colitis, while offspring from *Cldn2*KO and *Rag1*KO mice show resistance to colitis, though they die suddenly for unknown reasons (13). Together, these studies suggest a critical yet complex role of CLDN2 in colonic homeostasis and inflammation. Both epithelial and immune dysregulations underlie CAC susceptibility and progression (14, 15). Therefore, we wondered about the significance of CLDN2 upregulation in the susceptibility of patients with IBD to CAC, especially considering that CLDN2 is expressed at the crypt bottom; clusters with Cyclin-D1 and c-Myc proteins; and regulates intestinal epithelial cell (IEC) proliferation, differentiation, and migration as well as cancer stem cells in spontaneous colon cancer (CRC) (16, 17). Of note, CLDN2 expression is upregulated in and promotes CRC (16, 18–20).

In present study, we report the unexpected finding that *Cldn2*KO mice had significant inhibition of CAC despite severe colitis compared with WT littermates. Furthermore, we provide data that the protective role of CLDN2 against CAC stems from its currently unknown role in promoting MH. Our findings could increase current knowledge regarding the role of CLDN2 in IBD and clinical management of patients with IBD, including their disease progression and CAC risk.

## Results

### Loss of CLDN2 expression protects against CAC, despite promoting colitis.

**Figure 1:**
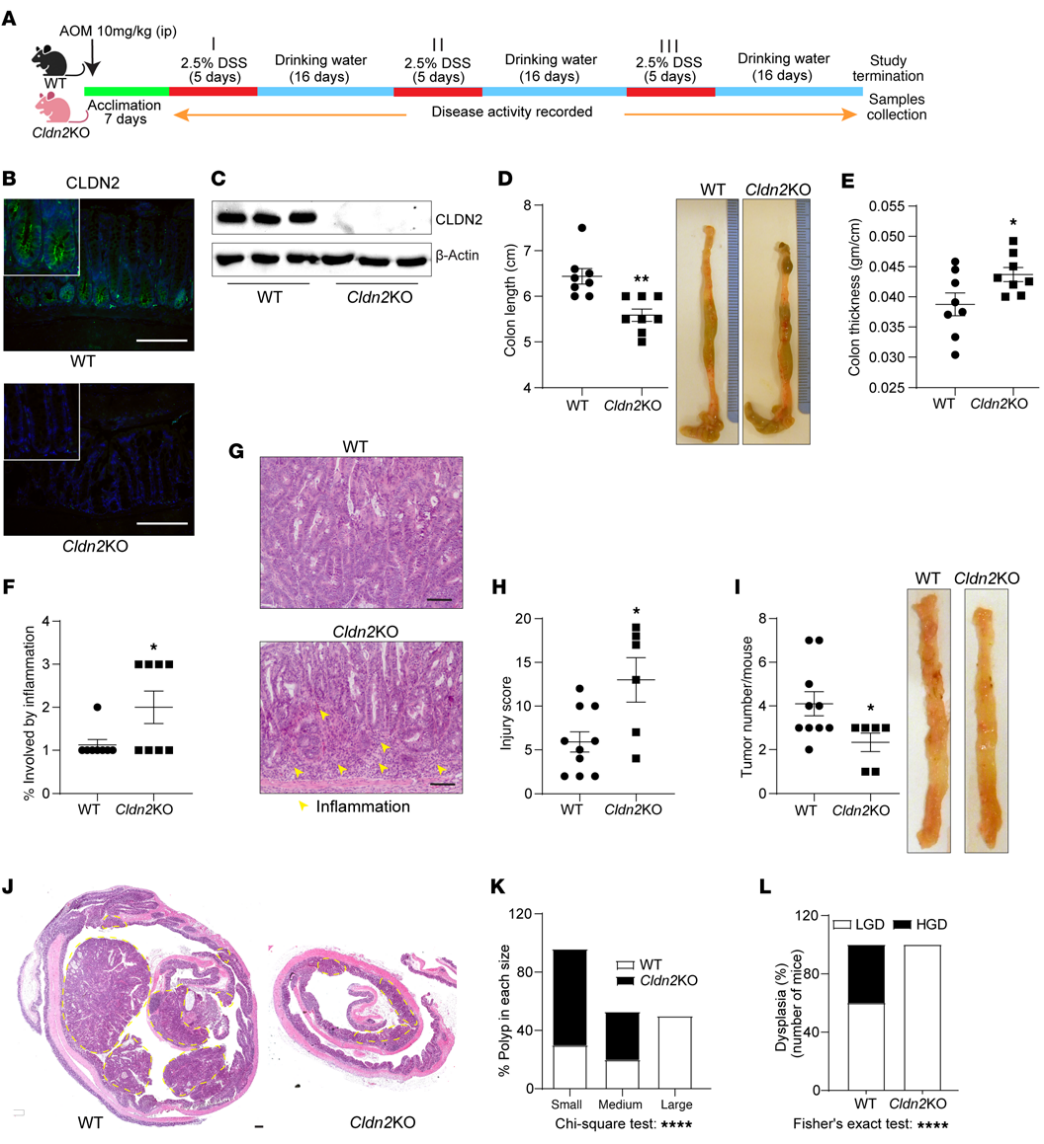
Colitis-associated cancer is significantly inhibited in *Cldn2*KO mice. (**A**) Schematic of the experimental strategy to induce colitis-associated colon cancer. (**B** and **C**) Representative immunofluorescence and immunoblot analysis confirming loss of CLDN2 expression in *Cldn2*KO mice. (**D**) Representative images of colons and colon length in AOM/DSS-treated *Cldn2*KO and WT mice (*n* = 8/group). (**E**) Colon edema (g/cm; *n* = 8/group). (**F**–**H**) Percentage of colon involved by inflammation (*n* = 8/group), representative H&E images, and mucosal injury score (WT/*Cldn2*KO: *n* = 10/6 mice). (**I** and **J**) Tumor growth and representative H&E analysis of the colons from AOM/DSS-treated *Cldn2*KO (*n* = 10) and WT (*n* = 6) mice. (**K** and **L**) Size of the colon polyps and dysplasia in *Cldn2*KO (*n* = 6) versus WT (*n* = 10) mice. Data in **D**–**F**, **H** and **I** are presented as the mean ± SEM. *P < 0.05, **P < 0.01 by 2-tailed unpaired *t* test. Data in **K** and **L** are presented as percentage number. ****P < 0.0001 by χ^2^ and Fisher’s exact test. Scale bar: 100 μM (**B** and **G**); 200 μM (**J**).

*Cldn2*KO mice showed, as reported, exacerbated colitis when subjected to DSS-induced colitis (Figure 1, B and C, and Supplemental Figure 1, A–C; supplemental material available online with this article; https://doi.org/10.1172/JCI170771DS1) (12, 21). We therefore hypothesized *Cldn2*KO mice to be susceptible to CAC. Interestingly, when subjected to azoxymethane (AOM)/DSS treatment, *Cldn2*KO mice showed a significantly lower tumor burden, despite higher colonic inflammation (Figure 1, A–K). In addition, tumors in *Cldn2*KO mice showed low-grade dysplasia compared with the high-grade dysplasia in WT mice (Figure 1L). Overall, above findings demonstrated an unexpected dichotomy in the effects of CLDN2 expression upon colitis and CAC.

### Cldn2 loss promotes proinflammatory and proapoptotic programs during CAC.

**Figure 2:**
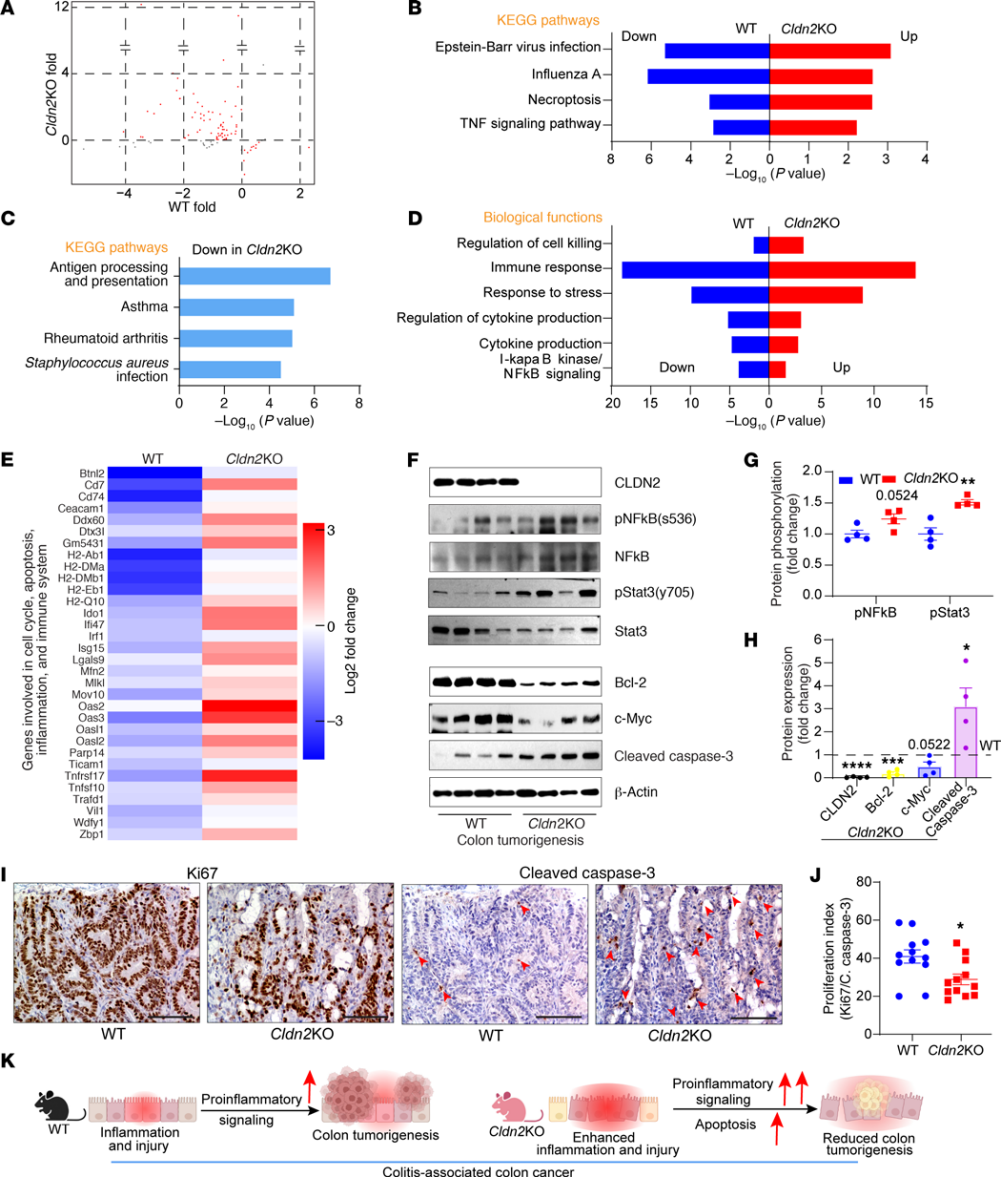
*Cldn2* loss-of-expression promotes proinflammatory and proapoptotic transcriptional programs in mice subjected to colitis-associated cancer. (**A**) Scatter plot depicting the comparative transcriptomic profile (RNA-Seq) between *Cldn2*KO and WT mice subjected to colitis-associated cancer (CAC) (*n* = 3/group). (**B**–**D**) KEGG pathway and GO biological function analysis based on differentially expressed genes (DEGs) in RNA-Seq analysis using colon RNA from AOM/DSS-treated *Cldn2*KO and WT mice (*n* = 3/group). (**E**) Heatmap showing selected DEGs (*n* = 3/group). (**F**–**H**) Immunoblotting and densitometric analysis of proteins involved in inflammation, proliferation, cell survival, and apoptosis in AOM/DSS-treated *Cldn2*KO and WT mice (*n* = 4/group). (**I** and **J**) Representative images of Ki67 and cleaved caspase-3 expression, and proliferation index (Ki67/cleaved caspase-3 expression) in AOM/DSS-treated mice (*n* = 4/group; 3 images/mice). (**K**) Graphical modeling representing the overall outcome of *Cldn2*KO mice when subjected to CAC. Data in **G**, **H**, and **J** are presented as the mean ± SEM. *P < 0.05, **P < 0.01, ***P < 0.001, ****P < 0.0001 by 2-tailed unpaired *t* test. Scale bar: 100 μM.

To understand why CLDN2 loss promotes colitis but not CAC, we examined global transcriptomic changes in CAC-challenged *Cldn2*KO colons versus WT mouse colons. Figure 2A shows the selected differentially expressed genes (DEGs). Notably, based on the Kyoto Encyclopedia of Genes and Genomes (KEGG) pathway analysis, prominent pathways upregulated in *Cldn2*KO mice included TNF and necroapoptosis. The downregulated pathways included antigen processing and presentation, asthma, and rheumatoid arthritis (versus WT mice). Gene Ontology (GO) analysis showed that the biological functions affected by the loss of CLDN2 also included NF-κB, stress and immune responses, and regulation of cell killing (Figure 2, B–D). The downregulated pathways included antigen processing and presentation and catabolic pathways (Supplemental Figure 1, D and E). The heatmap analysis further highlighted genes linked with the cell cycle, inflammation, apoptosis, and immune regulations in *Cldn2*KO mice versus WT mice (Figure 2E). Furthermore, immunoblotting and RT-qPCR analyses showed significant upregulation of pStat3^y705^, pNF-kB^s536^, IL-1, and IL-6 expression in AOM/DSS-treated *Cldn2*KO mice (Figure 2, F and G, and Supplemental Figure 1F). In addition, significant increases in the expression of the markers of DNA damage and apoptosis (γH2AX and cleaved caspase-3) and contrasting downregulation of cell survival and proliferation proteins (Bcl2 and c-Myc) characterized these mice (Figure 2, F and H, and Supplemental Figure 1, G–I). IHC analysis using antibodies against Ki67 and cleaved caspase-3 further showed a significant inhibition of the proliferative index (ratio of Ki67/cleaved caspase-3) in *Cldn2*KO mice (Figure 2, I and J). Overall, above data showed significant impairment of the crypt proliferation and survival programs in AOM/DSS-treated *Cldn2*KO mice despite higher inflammation (versus WT mice; Figure 2K).

### Colitis regulates CLDN2 expression in context-specific manner.

In light of above unexpected findings, we hypothesized a role for upregulated CLDN2 expression in IBD in promoting MH. We carefully examined CLDN2 regulation during colitis, as previous reports have shown variable CLDN2 expression in mouse models of colitis (10, 22). WT mice subjected to DSS-induced colitis, colitis/recovery, and chronic colitis were used (Supplemental Figure 2). Only distal colon, the principal site for the effects of DSS-induced colitis, was used (23). Interestingly, CLDN2 expression was downregulated in acute DSS-induced colitis colon samples although E-cadherin expression remained largely unaltered (Supplemental Figure 3A). Epithelium-enriched fractions from normal and colitis-subjected mouse colons showed similar outcomes (Supplemental Figure 3B). Coimmunofluorescence analysis for CLDN2 and β-catenin further showed robust β-catenin expression in inflamed colons lacking CLDN2, suggesting that it was not due to the loss of epithelium (Supplemental Figure 3C). Further analysis of the published microarray and RNA-Seq data sets from our and other laboratories from mice subjected to DSS-induced colitis validated *Cldn2* downregulation in colitis versus naive WT mice (Supplemental Figure 3, D and E) (10, 24). RT-qPCR also confirmed the downregulation of *Cldn2* expression in colitis (Supplemental Figure 3F).

**Figure 3:**
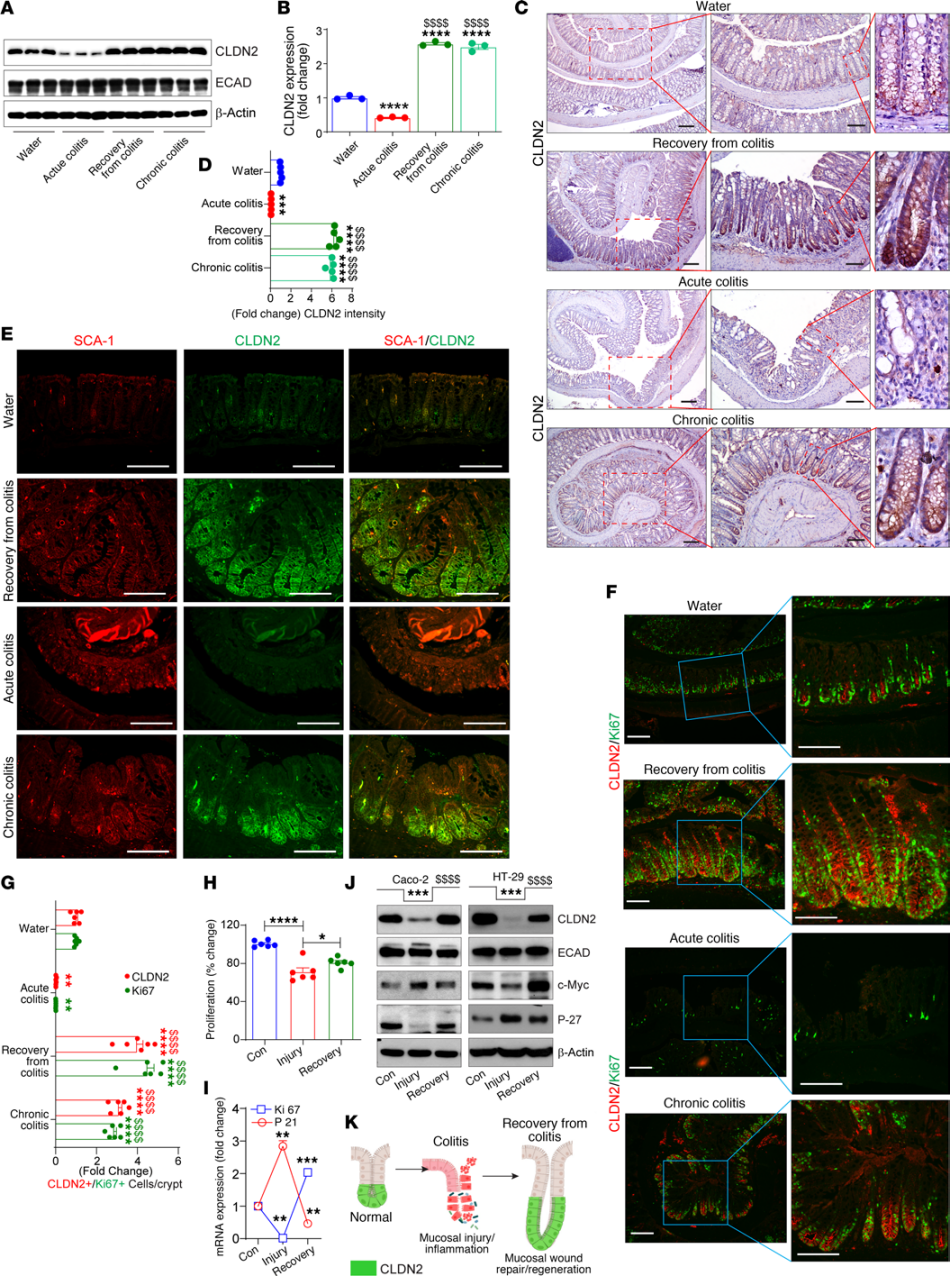
Colitis-mediated regulation of CLDN2 expression is context dependent and biphasic. (**A** and **B**) Epithelial enriched fractions of WT mouse colon that were untreated, subjected to DSS-induced colitis and recovery, or chronic DSS-induced colitis (*n* = 3/group). (**C** and **D**) Representative images and IHC intensity analysis using anti-CLDN2 antibody (*n* = 5/group). (**E**) Coimmunofluorescence image analysis for CLDN2 and SCA-1 in colon Swiss roll of mice subjected to acute DSS-induced colitis, DSS-induced colitis/recovery, or chronic DSS-induced colitis (*n* = 5/group). (**F** and **G**) Coimmunofluorescence and quantitative analysis using anti-CLDN2 and -Ki67 antibodies (*n* = 6/group). (**H** and **I**) MTT assay and RT-qPCR analysis using Caco2 cells subjected to DSS-induced injury and subsequent recovery (*n* = 3 independent experiments). (**J**) Immunoblot analysis for CLDN2, ECAD, c-Myc, and P27/Kip1 (*n* = 3 independent experiments). (**K**) Model depicting regulation of CLDN2 during colitis (injury phase) and recovery (repair/regeneration phase). Data in **B**, **D**, **G**, and **H**–**J** are presented as the mean ± SEM. *P < 0.05, **P < 0.01, ***P < 0.001, ****P < 0.0001, ^$$$$^*P* < 0.0001 by 1-way ANOVA with Tukey’s test. Scale bar: 100 μM (**C**, **E**, and **F**); 200 μM (**C**).

However, the above findings contrast with those found with an upregulated CLDN2 expression in IBD. Hence, we determined if it represents a response to the inflammation-associated injury. CLDN2 expression was examined in naive mice, mice subjected to DSS-induced colitis, colitis/recovery, or chronic-colitis. Interestingly, immunoblotting and IHC analyses showed a biphasic regulation of CLDN2 expression during colitis where CLDN2 expression was downregulated during DSS-induced acute colitis, but upregulated during recovery from colitis and in chronic colitis (Figure 3, A–D). We found similar CLDN2 upregulation in mice subjected to *C*. *rodentium* colitis (data not shown). Taken together, above data demonstrated dynamic regulation of CLDN2 expression during colitis where CLDN2 upregulation associated with MH.

### Colitis-associated CLDN2 upregulation correlates with crypt regeneration/repair.

In face of above findings, we further examined transcriptomic changes associated with CLDN2 changes during colitis-associated MH. High-throughput RNA-Seq analysis was performed using RNA isolated from the distal colons of mice subjected to recovery after DSS-induced colitis. The KEGG and GO analysis demonstrated that the biological processes involved in inflammation and apoptosis were negatively enriched in WT mice recovering from colitis while processes associated positively with repair/regeneration were upregulated (Supplemental Figure 4A). In contrast, during DSS-induced colitis, the proinflammatory and proapoptotic pathways were upregulated while the cell cycle pathways were inhibited (Supplemental Figure 3, D and E). We further found that *Cldn2* mRNA expression was downregulated during DSS-induced colitis; however, it was significantly upregulated during recovery along with *Pcna* and *Mki67ip* (markers of crypt proliferation). Expression of *Vil1* and *Cdkn1a* (associated with inhibition of cell growth) were downregulated (Supplemental Figure 3D and Supplemental Figure 4B).

In light of above outcomes, we further examined whether expression of stem cell antigen-1 (SCA-1), associated positively with mucosal repair/regeneration, is upregulated in mice subjected to colitis/recovery and its potential correlation with CLDN2 expression (25). Coimmunofluorescence analysis was done using anti-CLDN2 and -SCA-1 antibodies using colons from the mice subjected to colitis/recovery. As shown in Figure 3E, an upregulated SCA-1 expression characterized the regenerative crypts, which also colocalized with CLDN2 in mice subjected to DSS-induced colitis/recovery and/or chronic colitis. Additional analysis also demonstrated a significant upregulation for Ki67^+^ cells during recovery from colitis, which aligned positively with CLDN2^+^ cells in the regenerative epithelium of mice subjected to DSS-induced colitis/recovery and/or chronic colitis (vs. naive mice or mice subjected to acute colitis) (Figure 3, F and G).

To determine if effects described above on CLDN2 expression were epithelial intrinsic, we developed an ex vivo model of colitis-induced IEC injury/repair (Supplemental Figure 4C), based on published reports (26). Effects on the markers of cell proliferation/survival (MTT assay, Ki67 and c-Myc) and cell cycle inhibition (P-21/Cip1, and P-27/Kip1) supported distinct phases of injury and repair (Figure 3, H–J). As shown in Figure 3J, CLDN2 expression was downregulated during the injury phase; however, it was significantly upregulated during the repair/regeneration phase. E-cadherin expression remained unaltered. Similar findings in HT-29 cells showed that the effects were not cell specific (Figure 3J).

We further determined the association of CLDN2 with MH using colonoscope-associated wound healing. Immunohistochemical analysis of the colon 24 hours after wounding with colonoscope biopsy forceps demonstrated sharp upregulation of CLDN2 and Ki67 in the crypts adjacent to the wounds (Supplemental Figure 5). Above results supported an association of CLDN2 upregulation with intestinal epithelial injury/repair (Figure 3K).

### Cldn2KO mice show impaired recovery from colitis.

**Figure 4:**
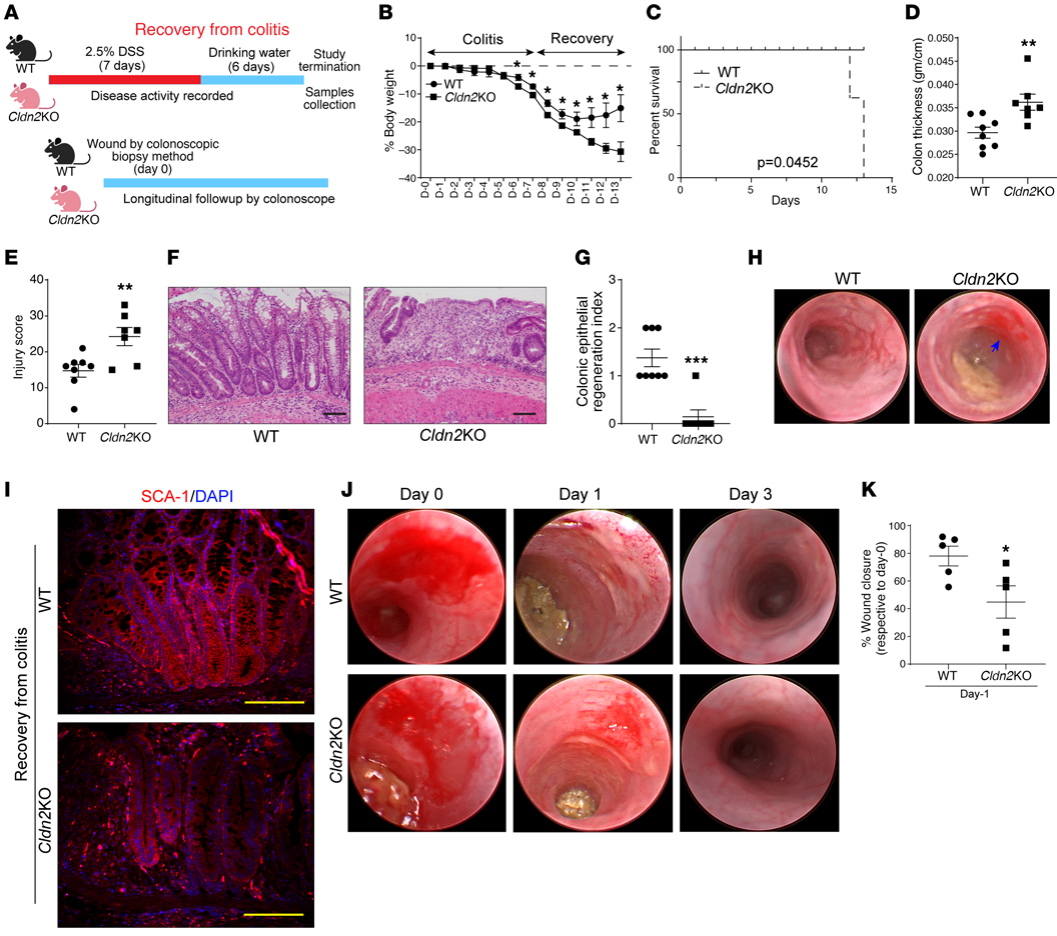
*Cldn2*KO mice show impaired recovery and mucosal healing following DSS-induced colitis. (**A**) Schematic illustration of experimental design. (**B**) Percentage weight change in *Cldn2*KO and WT mice subjected to DSS-induced colitis and recovery (*Cldn2*KO/WT: *n* = 8/7). (**C**) Km plot depicting mouse mortality during colitis/recovery (*Cldn2*KO/WT: *n* = 8/7). (**D**) Colon thickness (g/cm; *Cldn2*KO/WT: *n* = 8/7). (**E** and **F**) Mucosal injury score and representative H&E analysis (*Cldn2*KO/WT: *n* = 8/7). (**G**) Quantitation of epithelial regeneration by a pathologist in *Cldn2*KO and WT mice during recovery from colitis (*Cldn2*KO/WT: *n* = 8/7). (**H**) Representative images showing colonoscopic evaluation of colonic inflammation in *Cldn2*KO mice compared with WT mice. (**I**) Coimmunofluorescence image analysis for SCA-1 in colon Swiss roll of *Cldn2*KO and WT mice subjected to recovery from colitis. (**J**) Wound healing assay using colonoscope-assisted wounding. (**K**) Quantitative image analysis shows delayed mucosal healing in *Cldn2*KO mice compared with that in WT mice (*n* = 5/group). Data in **B**, **D**, **E**, **G**, and **K** are presented as the mean ± SEM. *P < 0.05, **P < 0.01, ***P < 0.001 by 2-tailed unpaired *t* test. Survival data in **C** was assessed by log-rank (Mantel-Cox) test. Scale bar: 100 μM.

To further determine a causal role for CLDN2 in colitis-associated MH, we subjected *Cldn2*KO mice and littermate WT mice to DSS-induced colitis and recovery (Figure 4A). As shown in Figure 4B, DSS treatment significantly lowered the body weight of WT mice, which reverted to the normal during the recovery. *Cldn2*KO mice exhibited significantly higher weight loss during colitis and further failed to recover the weight loss during recovery (Figure 4B). In addition, *Cldn2*KO mice died during recovery (Figure 4C). Colon thickness and mucosal injury were significantly high in *Cldn2*KO mice (versus WT mice; Figure 4, D and E). Histopathological analysis further revealed increased immune cell infiltration and a significant reduction in the regenerative potential (Figure 4, F and G). Colonoscope-assisted longitudinal mapping further supported progressive worsening of colitis in *Cldn2*KO mice (Figure 4H). In addition, expression of SCA-1 was significantly downregulated in *Cldn2*KO mice recovering from DSS-induced colitis (Figure 4I).

We also examined relative healing of the colonoscope-assisted wounding in WT and *Cldn2*KO mice (Figure 4A). As shown in Figure 4, J and K, WT mice showed a substantial reduction in the ulcerated area on day 1. By day 3, the original lesion was almost indistinguishable from the non-injured colon. In *Cldn2*KO mouse colons, the reddish appearance of the tissue indicated incomplete healing (Figure 4, J and K). Overall, our findings demonstrated that the loss of CLDN2 expression impairs recovery/regeneration from mucosal injury.

### Loss of Cldn2 expression promotes proinflammatory and proapoptotic responses during recovery from colitis.

**Figure 5:**
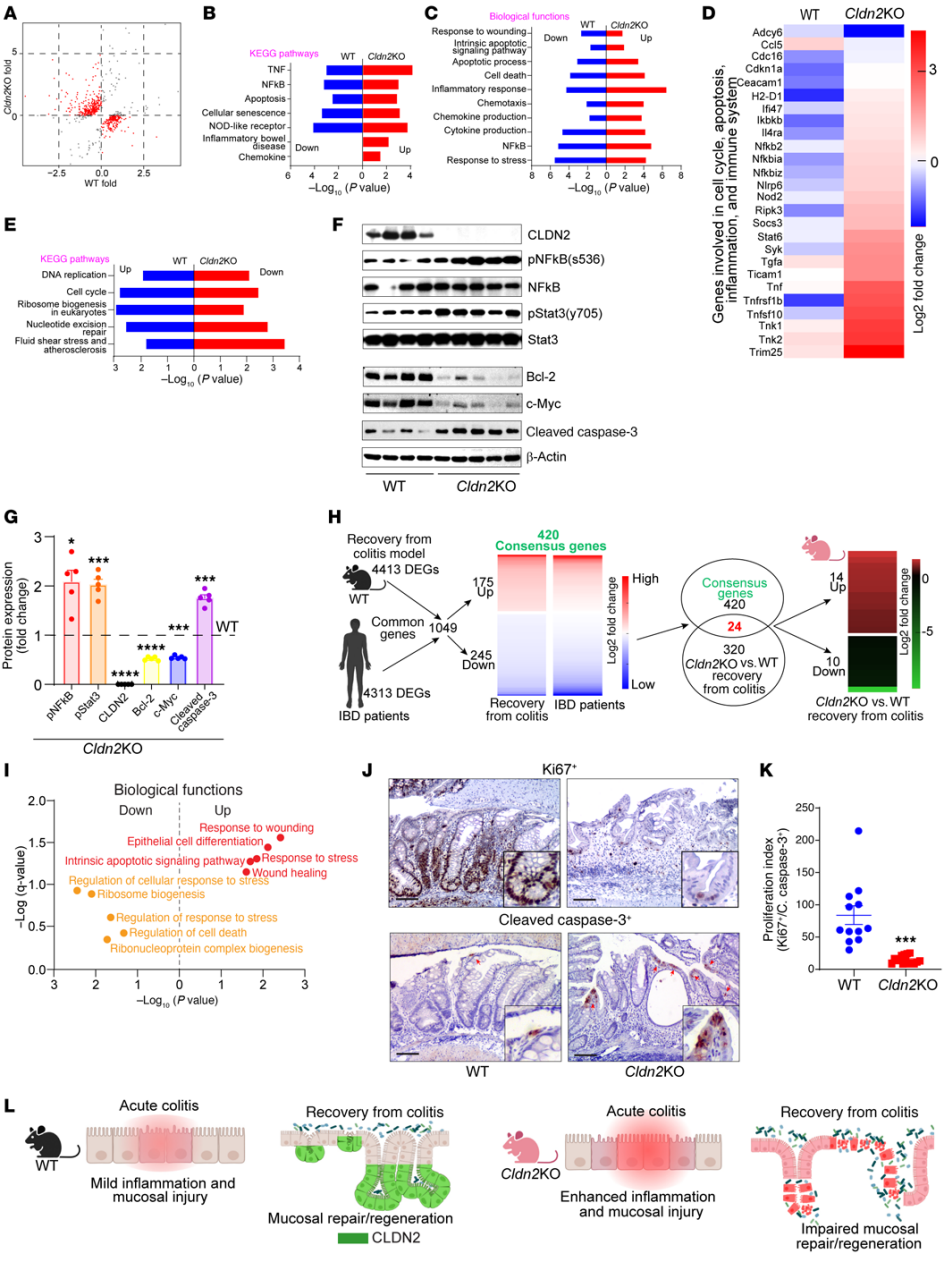
Loss of *Cldn2* results in a defective mucosal healing transcriptomic response, which differs from the conserved gene expression profile between WT mice recovering from IBD and patients with IBD. (**A**) Scatter graph showing differential gene expression between *Cldn2*KO and WT mice recovering from colitis (*n* = 3/group). (**B** and **C**) Most prominent KEGG pathways and GO biological processes based on gene expression upregulated in *Cldn2*KO mice versus WT mice during recovery from colitis (*n* = 3/group). (**D**) Heatmap depicting differential gene expression, which was primarily associated with cell cycle, apoptosis, inflammation, and immune homeostasis (*Cldn2*KO versus WT mice; *n* = 3/group). (**E**) Most prominent downregulated KEGG pathways in *Cldn2*KO mice compared with WT mice (*n* = 3/group). (**F** and **G**) Immunoblotting and densitometric analysis of proteins involved in inflammation, proliferation, survival, and apoptosis in mice recovering from colitis (*Cldn2*KO; *n* = 5) and WT (*n* = 4) mice. (**H**) The gene profile in *Cldn2*KO mice recovering from colitis differs from the conserved profile between mice and humans. (**I**) Significant dysregulation of GO biological processes that are associated with mucosal healing in *Cldn2*KO mice versus WT mice. (**J** and **K**) Immunohistochemical analysis for cleaved caspase-3 and Ki67, and proliferation index (WT/*Cldn2*KO: *n* = 4/5 mice; 3 fields in each mice Swiss role). (**L**) Schematics showing that loss of CLDN2 results in impaired mucosal healing. Data in **G** and **K** are presented as the mean ± SEM. *P < 0.05, ***P < 0.001, ****P < 0.0001 by 2-tailed unpaired *t* test. Scale bar: 100 μM.

To determine why *Cldn2*KO mice show impaired recovery from colitis, we performed RNA-Seq analysis. The volcano plot (Figure 5A) shows contrasting transcriptomes in *Cldn2*KO versus WT mice based on the Euclidean distance between the DEGs (log_2_ fold change; *P* < 0.05). The top upregulated pathways in *Cldn2*KO mice (KEGG pathway analysis) were the proinflammatory and apoptotic pathways (Figure 5, B and D). The top biological functions (GO analysis) included the apoptotic process, cell death, cytokine production, and response to wounding, etc. (versus WT; Figure 5, C and D). In accordance, we found increased expression of IL-1 and IL-6 (Supplemental Figure 6A) as well as pNF-kB^s536^ and pStat3^y705^ in *Cldn2*KO mice recovering from colitis (Figure 5, F and G). The downregulated pathways in *Cldn2*KO mice included DNA replication and cell cycle (Figure 5, D and E). The metabolic related processes were also downregulated in *Cldn2*KO mice (Supplemental Figure 6B). Immunoblotting analysis further supported dysregulation of the DNA damage response (DDR) in *Cldn2*KO mice. In this regard, expression of γH2AX, P-21/Cip1 and pChk2^t68^, proteins associated with DDR, was significantly upregulated in *Cldn2*KO mice when subjected to recovery from DSS-induced colitis (vs. WT mice; Supplemental Figure 6, C and D). Immunoblotting using the same samples further showed significant downregulation of Bcl2 and C-Myc expressions in *Cldn2*KO mice while cleaved caspase-3 expression was upregulated (Figure 5, F and G). The above findings strongly support a role for CLDN2 expression in colitis-associated epithelial cell injury and thus restitution/repair.

### CLDN2 deficiency associates with impaired MH-associated gene transcription in both colitis-challenged mice and patients with IBD.

Having characterized that *Cldn2* loss promotes proinflammatory and proapoptotic programs during colitis/recovery, we further examined its relevance to IBD. To this end, we compared the DEGs from WT mice subjected to recovery from colitis with DEGs in patients with IBD versus healthy humans (27). Among 4,413 DEGs from mice, 1,049 genes overlapped with the DEGs in patients with IBD. Of these, 420 genes had a similar tendency of regulation in mice recovering from colitis and patients with IBD (Figure 5H). Further determination of the potential overlap between these shared DEGs (colitis-challenged mice and patients with IBD) and the DEGs in *Cldn2*KO mice versus WT mice (recovering from colitis) identified 24 genes (14 up- and 10 downregulated; Figure 5H and Supplemental Table 1). The GO analysis, based on these 14 upregulated DEGs, identified response to wounding, wound healing, epithelial cell differentiation, and intrinsic apoptotic signaling. The downregulated biological functions included regulation of the cellular response to stress and cell death (Figure 5I). Furthermore, proliferative index, based on the ratio of proliferation/apoptosis, was significantly downregulated in *Cldn2*KO versus WT mice (Figure 5, J and K). Additional analysis of our published microarray data set for the DEGs in Villin-*Cldn2*TG versus WT mice (DSS-induced colitis) revealed contrasting upregulation of the cell proliferation pathways and downregulation of the inflammatory and apoptotic pathways (Supplemental Figure 7, A and B) (10). Overall, these results supported a role for CLDN2 in promoting colitis-associated MH (Figure 5L).

### Inhibiting EGFR signaling inhibits recovery from colitis and associated CLDN2 upregulation.

**Figure 6:**
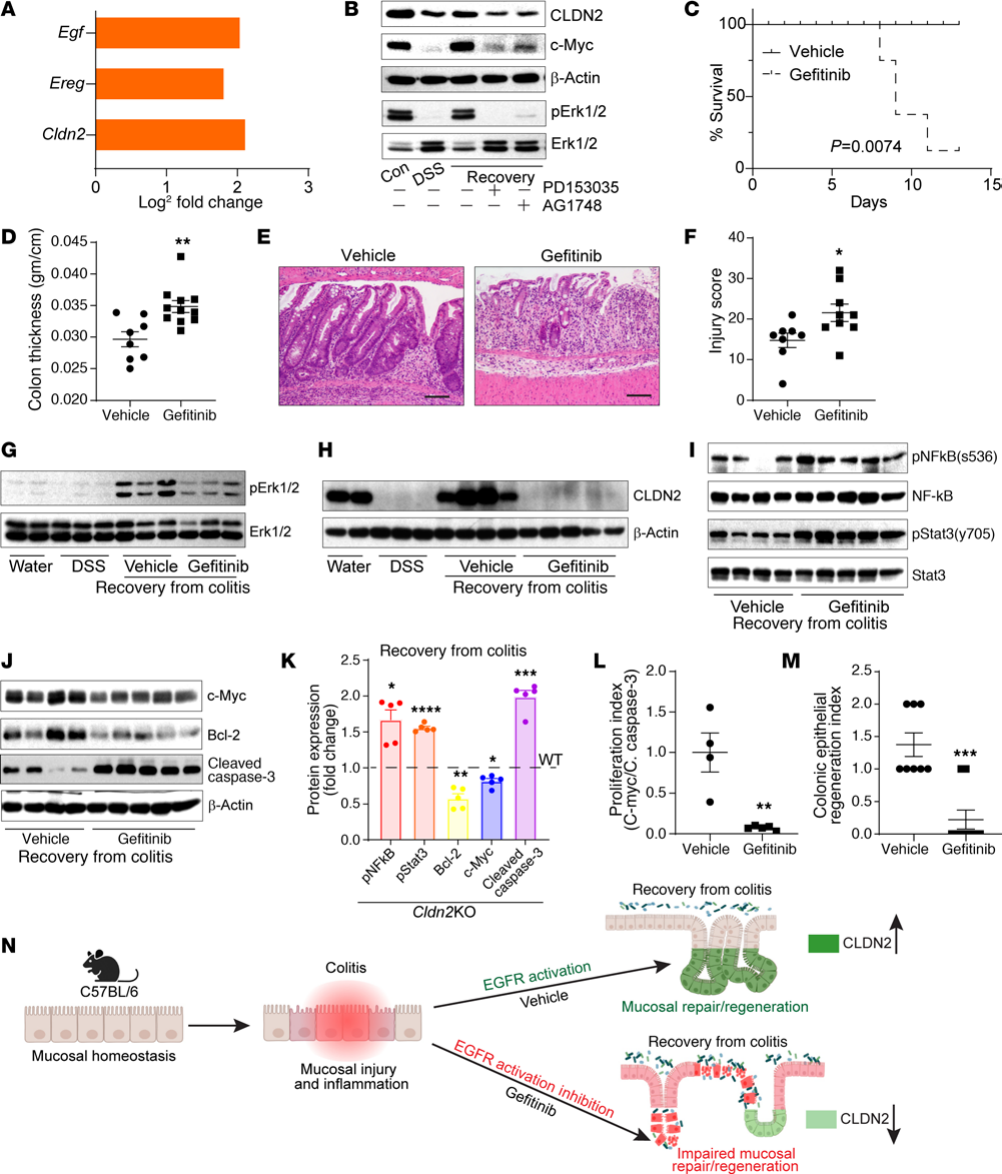
Inhibiting EGFR signaling in WT mice recovering from DSS-induced colitis inhibits CLDN2 upregulation and impairs mucosal healing. (**A**) Differentially expressed genes (DEGs; RNA-Seq analysis) in mice recovering from DSS-induced colitis show upregulated *Cldn2* expression, along with *Egf* and *Ereg* (EGFR ligands) (*n* = 3/group). (**B**) Immunoblot analysis using Caco-2 cells subjected to DSS-induced injury/repair with or without inhibitors of EGFR activation (*n* = 3 independent experiments). (**C**) Km analysis for survival in mice receiving EGFR inhibitor (gefitinib) during recovery from colitis (vehicle/gefitinib: *n* = 9/13). (**D**) Colon thickness (g/cm; vehicle/gefitinib: *n* = 8/11). (**E**) H&E images showing profound mucosal injury/impaired regeneration in gefitinib-treated mice and (**F**) mucosal injury score (vehicle/gefitinib: *n* = 8/9). (**G** and **H**) Immunoblot analysis using total colon lysate. pErk1/2/Erk1/2 and CLDN2 expression served as a marker of EGFR activation (water/DSS/vehicle/gefitinib: *n* = 2/3/3/3). (**I**–**K**) Immunoblot analysis for the markers of inflammation, cell survival, and apoptosis in gefitinib- and vehicle-treated mice during recovery from colitis (vehicle/gefitinib: *n* = 4/5). (**L**) Proliferative index (vehicle/gefitinib: *n* = 4/5). (**M**) Epithelial regenerative index in gefitinib-treated mice versus vehicle-treated WT mice (vehicle/gefitinib: *n* = 8/9). (**N**) Graphical summary depicting integration between EGFR and CLDN2 in colitis-associated epithelial restitution/healing. Survival data in **C** were assessed by log-rank (Mantel-Cox) test. Data in **D**, **F**, and **K**–**M** are presented as the mean ± SEM. *P < 0.05, **P < 0.01, ***P < 0.001, ****P < 0.0001 by 2-tailed unpaired *t* test. Scale bar: 100 μM.

Having uncovered a potentially novel role for CLDN2 upregulation in inflammation-associated MH, we wondered about its pathological significance in face of the prosurvival signaling that promote MH (18, 28–30). EGFR signaling promotes colonic CLDN2 expression (18, 31). EGFR activation protects patients with IBD from colitis (29, 32, 33). Furthermore, our RNA-Seq analysis revealed significant increases in *Egf* and *Ereg* expression, ligands that activate EGFR signaling, along with *Cldn2* (Figure 6A). Hence, we examined if colitis-induced CLDN2 upregulation depends on EGFR activation. Caco-2 cells recovering from DSS injury were subjected to the inhibitors of EGFR activation. Inhibiting EGFR signaling inhibited CLDN2 upregulation along with c-Myc and pERK1/2 expression (Figure 6B). To examine this in vivo, we inhibited EGFR signaling (gefitinib; 200 mg/kg body weight; oral gavage) in WT mice recovering from DSS-induced colitis. Inhibiting EGFR activation impaired recovery from colitis and resulted in mouse death (Figure 6, C and D). Histological evaluation showed increased inflammation, mucosal injury, and edema in EGFR-inhibited mice versus untreated mice (Figure 6, E and F). Inhibition of pERK1/2 supported the efficacy of the inhibition of EGFR activation (Figure 6G). Notably, recovery-associated CLDN2 upregulation was also inhibited in EGFR-inhibited mice (Figure 6H). Inhibiting EGFR signaling in mice recovering from colitis also promoted expression of pNF-κB^s536^ and pStat3^y705^ (Figure 3, I and K) while the proliferative index was significantly lower (Figure 6, J–L), similar to that in *Cldn2*KO mice. The regenerative index was also significantly lower in gefitinib-treated mice (Figure 6M). These results supported a critical role for CLDN2 expression in EGFR-mediated regulation of inflammation-associated MH (Figure 6N).

### Colonic CLDN2 upregulation, as in IBD, protects against CAC.

**Figure 7:**
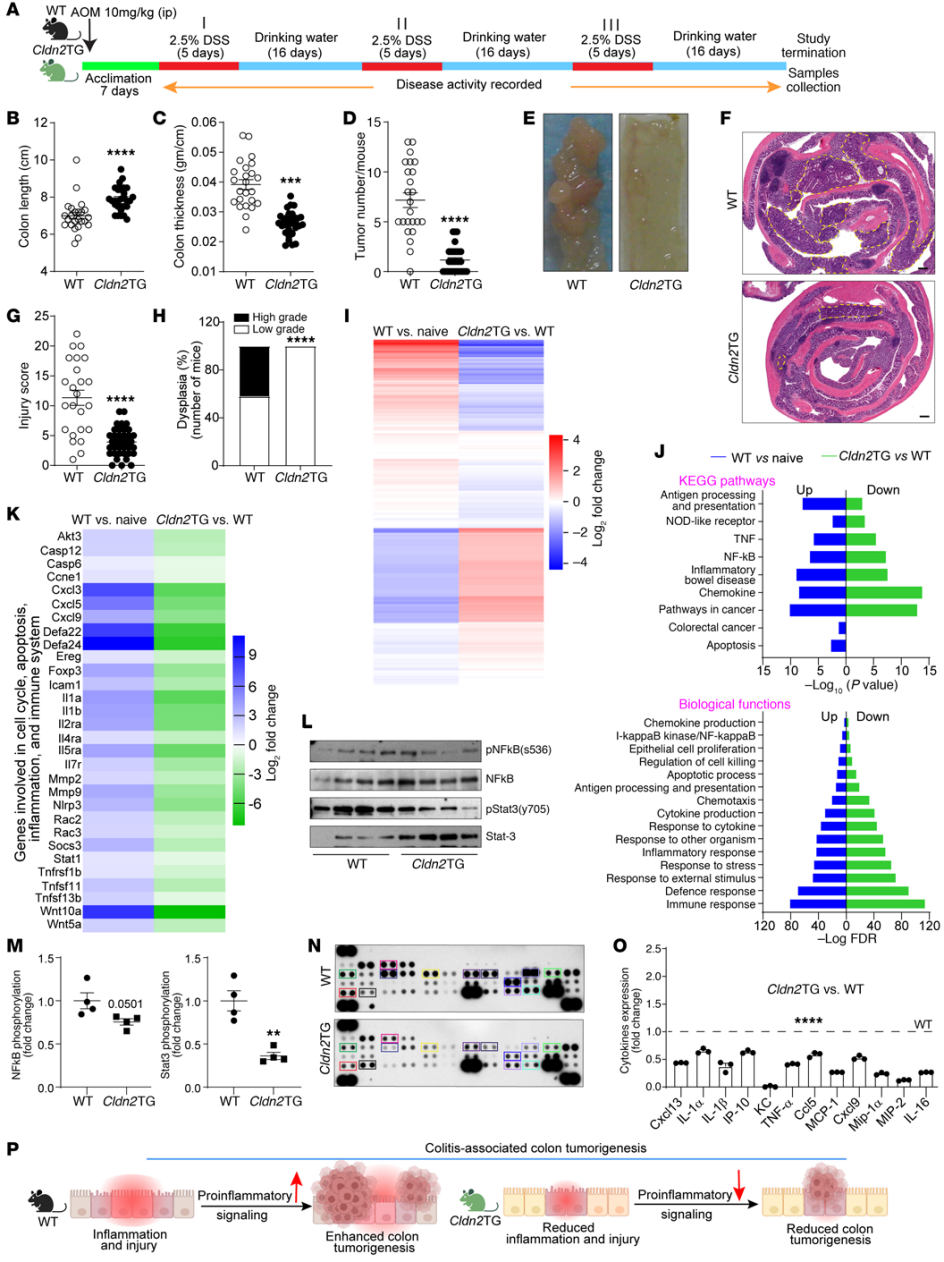
CLDN2 overexpression in the intestinal epithelium protects mice from colitis-associated cancer. (**A**) Graphical illustration of AOM/DSS tumorigenesis in Villin-*Cldn2*TG and WT mice. (**B**) Colon length (cm; WT/Villin-*Cldn2*TG: *n* = 24/31). (**C**) Colon edema (g/cm; WT/Villin-*Cldn2*TG: *n* = 24/31). (**D** and **E**) Tumor growth in the colon and representative images of the AOM/DSS-treated Villin-*Cldn2*TG and WT mouse colon (WT/Villin-*Cldn2*TG: *n* = 24/31). (**F** and **G**) H&E analysis and mucosal injury score (WT/Villin-*Cldn2*TG: *n* = 24/37). (**H**) Percentage of dysplasia in mice treated with AOM/DSS. (**I**) RNA-Seq analysis (differentially regulated genes [DEGs]) in mice subjected to AOM/DSS treatment (*n* = 3/group). (**J**) Significantly altered KEGG pathways and GO biological function in AOM/DSS-treated Villin-*Cldn2*TG and WT mice (*n* = 3/group). (**K**) Heatmap depicting the DEGs associated with cell cycle, apoptosis, inflammation, and immune homeostasis (*n* = 3/group). (**L** and **M**) Immunoblotting and densitometric analysis for Stat3 and NF-κB activation in mice subjected to AOM/DSS (*n* = 4/group). (**N** and **O**) High-throughput analysis for cytokine expression using total colon lysate and densitometry evaluations (*n* = 3/group). (**P**) Cartoon summarizing the role of upregulated CLDN2 in CAC. Data in **B**–**D**, **G**, and **M** are presented as the mean ± SEM. **P < 0.01, ***P < 0.001, ****P < 0.0001 by 2-tailed unpaired *t* test. Data in **H** are presented as percentage number. ****P < 0.0001 by Fisher’s exact test. Data in **O** are presented as the mean ± SEM. ****P < 0.0001 by 1-way ANOVA with Dunnett’s multiple comparisons. Scale bar: 200 μM.

In light of the above findings, we further determined the significance of the IBD-associated CLDN2 upregulation in the susceptibility to CAC. Villin-*Cldn2*TG mice were subjected to AOM/DSS treatment (Figure 7A) (31). Masked histological (H&E) analysis and scoring of colonic inflammation and injury demonstrated a significant decrease in proinflammatory responses in *Cldn2*TG mice (versus WT mice; Figure 7, B–G). In addition, tumors in *Cldn2*TG mice were less aggressive compared with those in WT mice (Figure 7H). RNA-Seq analysis for DEGs further revealed significant downregulation of proinflammatory genes/pathways in CAC-subjected *Cldn2*TG mice (versus WT mice; Figure 7, I–K). Immunoblot and multiplex cytokine analysis using total colon lysates further showed significant downregulation of proinflammatory cytokines in *Cldn2*TG mice (versus WT mice; Figure 7, L–O). Overall, these data suggested an unexpected role of CLDN2 upregulation in ameliorating the susceptibility to CAC (Figure 7P).

### CLDN2 expression modulates Survivin expression to regulate crypt cell apoptosis.

**Figure 8:**
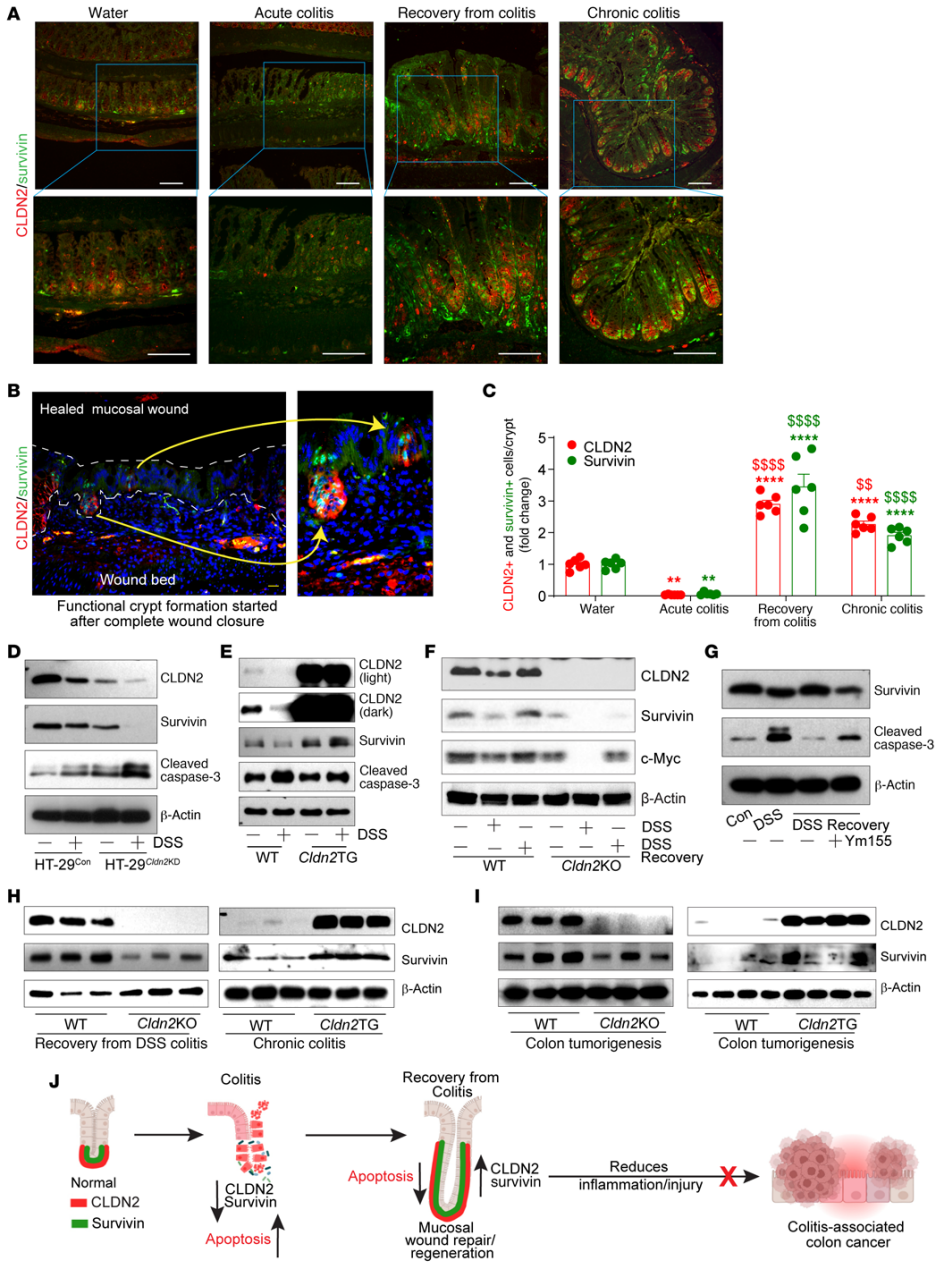
CLDN2 expression promotes colitis-associated epithelial repair/regeneration in Survivin-dependent manner. (**A**–**C**) Coimmunofluorescence and quantitative analysis for CLDN2 and Survivin expression in WT mice recovering from DSS-induced colitis (*n* = 6/group). (**D** and **E**) Immunoblot analysis using lysates from the DSS-treated colonic epithelial cells and ex vivo 3D culture of the colon crypts from control and *Cldn2*KD cells and WT and Villin-*Cldn2*TG mice (*n* = 3 independent experiments). (**F**) Immunoblots analysis using lysate from colon epithelial cells of *Cldn2*KO and WT mice subjected to the DSS-induced injury/repair (*n* = 3 independent experiments). (**G**) Caco-2 cells subjected to DSS-induced injury/repair with or without inhibitors of the Survivin(*n* = 3 independent experiments). (**H** and **I**) Immunoblotting and densitometry analysis of Survivin in *Cldn2*KO (*n* = 3) and Villin-*Cldn2*TG (*n* = 3) mice versus WT (*n* = 3 and 3) mice subjected to recovery from colitis, chronic colitis, and CAC tumorigenesis (WT/*Cldn2*KO: *n* = 3/3; WT/Villin-*Cldn2*TG: *n* = 4/4). (**J**) Schematic illustration depicting the role of CLDN2 and Survivin in mucosal repair/regeneration. Data in **C** are presented as the mean ± SEM. **P < 0.01, ^$$^*P* < 0.01, ****P < 0.0001, ^$$$$^*P* < 0001 by 1-way ANOVA with Tukey’s test. Scale bar: 100 μM (**A**); 200 μM (**B**).

Epithelial cell apoptosis is linked with mucosal damage and disease severity in IBD. MH depends on the ratio of the antiapoptotic to proapoptotic proteins (34–36). Survivin, a member of the inhibitor of apoptosis family, plays a key role in colitis-associated crypt regeneration (37). Hence, we examined whether CLDN2 regulates Survivin expression to regulate colitis-associated MH. Coimmunofluorescence analysis using anti-CLDN2 and -Survivin antibody showed a positive correlation in colitis-associated regenerative crypts (Figure 8, A–C). We further examined whether Survivin expression changes associate with CLDN2 expression changes irrespective of the mode of intestinal injury. We used γ-irradiation and 5-fluorouracil (5FU) as the source of intestinal injury, using both in vivo and ex vivo models. In both, γ-irradiated colon crypts and Caco-2 cells, we found downregulation of CLDN2 along with decreases in Survivin and c-Myc expression (Supplemental Figure 8, A–D). Expression of cleaved PARP was upregulated (Supplemental Figure 8, A–D). We found a similar downregulation of CLDN2 and Survivin expression in mouse colons and Caco-2 cells when subjected to the 5FU treatment (Supplemental Figure 8, E–H). To further examine the causal role of CLDN2 in modulating Survivin expression, we used HT-29^Cldn2KD^ cells in which CLDN2 expression was stably inhibited. Ex vivo culture of colon crypts from *Cldn2*TG and WT mice was also used and subjected to DSS-induced injury. Immunoblot analysis showed a linear association between CLDN2 and Survivin expression, which contrasted with expression of cleaved caspase-3 (Figure 8, D and E, and Supplemental Figure 8, I and J). We further validated this causal association using epithelial cells isolated from the intestinal crypts from *Cldn2*KO and WT mice as well as control and HT29^Cldn2KD^ cells subjected to the DSS injury and repair. Immunoblot analysis supported a causal association of CLDN2 expression in regulating Survivin expression during intestinal epithelial injury/repair (Figure 8F and Supplemental Figure 8, K–M). To complement this, Cldn2overexpressing cells were subjected to DSS-induced injury/recovery with and without inhibition of Survivin (Ym155) during the recovery phase. As shown in Figure 8G and Supplemental Figure 8N, despite sustained CLDN2 overexpression, inhibiting Survivin signaling promoted cleaved caspase-3 expression. Taken together, above data suggested a causal role of CLDN2 in modulating Survivin expression during injury/repair.

### The CLDN2/Survivin axis helps promotes MH and may inhibit progression of colitis to CAC.

We next tested the significance of the gain or loss of function of the CLDN2/Survivin axis in colitis-associated repair/MH. Immunoblot analysis using lysates from *Cldn2*KO mice subjected to recovery from DSS-induced colitis showed downregulated Survivin expression (versus WT mice). In contrast, Survivin expression was significantly upregulated in *Cldn2*TG mice subjected to chronic colitis (Figure 8H and Supplemental Figure 8O). We have previously reported that *Cldn2*TG mice are protected from chronic DSS-induced colitis (10). A recent report suggests that Survivin expression is upregulated during the resolution of inflammation and CAC (37). We therefore further sought to specify the role of the CLDN2/Survivin axis using both *Cldn2*KO and *Cldn2*TG mice during CAC development. As shown in Figure 8I and Supplemental Figure 8P, we found a context-specific role of the CLDN2/Survivin axis during CAC development. The overall postulation based on above data is illustrated in Figure 8J.

### An upregulated CLDN2 expression associates positively with Survivin expression and MH in patients with IBD.

**Figure 9:**
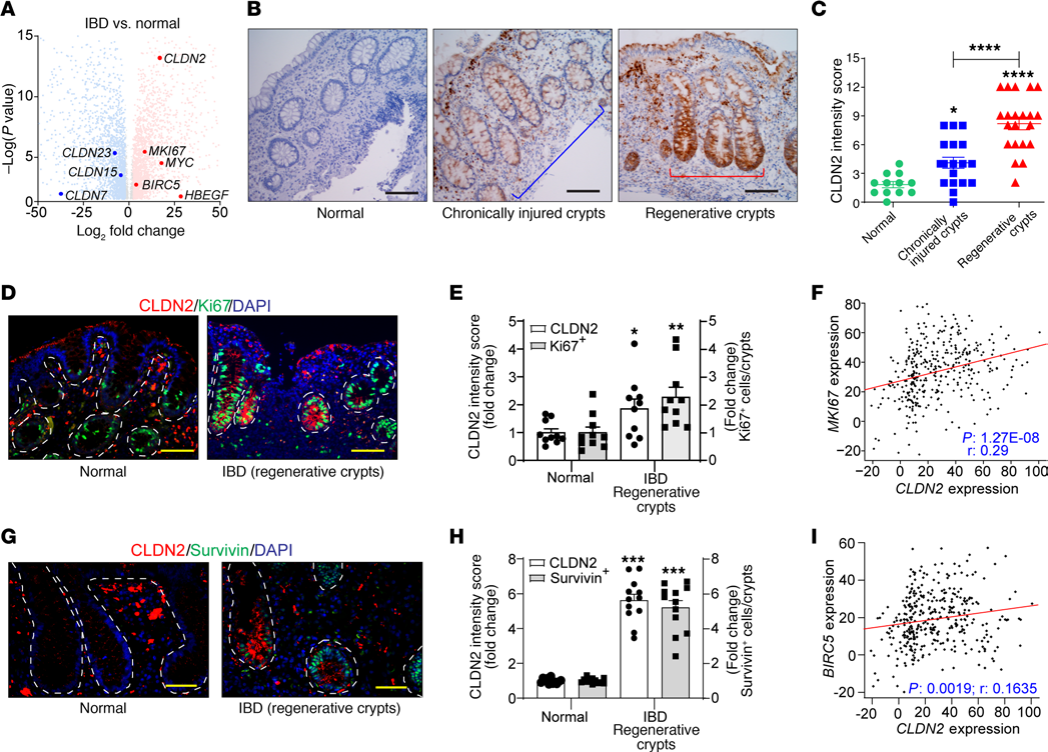
In patients with IBD, colitis-associated CLDN2 upregulation is concentrated primarily in the regenerative crypts and correlates with Ki67 and Survivin expression. (**A**) In silico analysis of published transcriptome data from patients with IBD (55–65) (*n* = 360) demonstrating significantly upregulated CLDN2 expression compared with that in normal individuals (*n* = 153). (**B** and **C**) Immunohistochemical analysis of CLDN2 expression in biopsy samples from patients with IBD versus normal colon and intensity scoring. The pathological evaluation demonstrated significantly higher CLDN2 expression in the regenerative crypts (normal: *n* = 12; chronically injured crypts: *n* = 18; regenerative crypts: *n* = 21). (**D**–**F**) Coimmunofluorescence analysis of CLDN2 and Ki67 expression, along with in silico correlation analysis between *CLDN2* and *MKI67* in published data sets from patients with IBD (55–65) (*n* = 10/group). (**G** and **H**) Survivin and CLDN2 expression in IBD biopsy samples compared with normal samples (12/group). (**I**) In silico analysis of the correlation between *CLDN2* and *BIRC5* expression in published data sets from patients with IBD (55–65) (normal/IBD: *n* = 153/360). Data in **C** are presented as the mean ± SEM. *P < 0.05, ****P < 0.0001 by 1-way ANOVA with Tukey’s test. Data in **E** and **H** are presented as the mean ± SEM. *P < 0.05, **P < 0.01, ***P < 0.001 by 2-tailed unpaired *t* test. Pearson’s correlation analysis was performed in **F** and **I**. Scale bar: 100 μM.

To determine the clinical significance of our findings, we further examined CLDN2 expression in patients with IBD in association with MH. We first performed an unbiased in silico analysis of the published high-throughput transcriptome from patients with IBD (see Methods). Quantitative assessment demonstrated a robust *CLDN2* upregulation in patients with IBD versus healthy individuals; however, there was also downregulation of other claudins (Figure 9A). An upregulated CLDN2 expression also characterized the biopsy samples from patients with IBD (Figure 9B). Notably, the blinded determination by a GI pathologists determined that CLDN2 expression in such biopsies was associated positively with the regenerative crypts compared with the adjacent chronically injured crypts (Figure 9, B and C). Coimmunofluorescence and in silico analysis using samples from patients with IBD and a data set further validated a positive association of CLDN2 expression with Ki67 (Figure 9, A and D–F). Our analysis of cohorts of patients with IBD further revealed a significant increase in *BIRC5* (Survivin) expression and a positive association with *CLDN2* expression (Figure 9, A and G–I). Taken together, the above data support a potentially novel role of CLDN2 in regulating Survivin signaling to promote inflammation-associated regeneration/repair and thus delay the CAC progression.

## Discussion

CAC is a comorbidity of IBD and a major cause of patient death (38). Our current findings reveal a role for CLDN2 expression in protecting against CAC through its potentially novel role in promoting MH. A dysregulated balance between proliferation and apoptosis is central to the oncogenic growth, and our data suggest that the suppressed CAC in *Cldn2*KO mice despite chronic inflammation is due to increased IEC apoptosis. In this regard, inflammation/injury-associated CLDN2 upregulation was restricted to the SCA-1^+^, Ki67^+^, and/or Survivin^+^ cells, and *Cldn2* loss impaired the recovery of mice from colitis. It is noteworthy here that the proinflammatory signaling and molecular apparatus, known to promote IEC death and that characterizes the WT mice subjected to acute colitis, persisted in *Cldn2*KO mice, even when they were allowed to recover from colitis. Our additional finding that SCA-1 and Survivin expression is co-upregulated with CLDN2 in the regenerating crypts in WT mice but not in *Cldn2*KO mice aligns with our overall hypothesis, as both SCA-1 and Survivin signaling promote regeneration/repair (37, 39, 40).

Importantly, intestinal CLDN2 expression is uniquely restricted to the crypt base, among claudin proteins expressed in intestine and among leaky and proliferative colonocytes (10, 18, 41). Furthermore, intestinal *Cldn2* expression decreases with age and maturation of the gut barrier (42). These findings led to an appreciation of CLDN2 upregulation in IBD and its role in gut permeability (8, 9, 43). However, mouse modeling of CLDN2 expression, as in IBD, unexpectedly protected from colitis (10, 11). The contrasting increase in colitis in *Cldn2*KO mice suggested a rather adaptive role of colitis-induced CLDN2 upregulation (12). A recent report that depleting the T and B lymphocytes in Villin-*Cldn2*TG mice renders them susceptible to T cell colitis contrasts above findings; however, it also suggests a direct/indirect role of CLDN2 in mucosal immune homeostasis (13). In this regard, we have previously reported an immune-suppressive phenotype in *Cldn2*TG mice (10). A contrasting increase in proinflammatory signaling characterized *Cldn2*KO mice when subjected to colitis, which we have validated in current studies (12, 44). An explanation for such immune-suppressive effects of CLDN2 upregulation could be its role in mucosal Na^+^ (paracellular) transport and hence water flux, as described previously (11). However, a report that CLDN2 expression is dispensable for mucosal Na^+^ homeostasis argues against this postulation (45, 46). Moreover, Raju et al. found that compensatory upregulation of CLDN15, a paracellular cation transporter, in colitis-challenged *Rag-1*KO/*Cldn2*KO mice was insufficient to rescue the disease phenotype (13). Notably, claudin-15 expression is indispensable for intestinal Na^+^ transport (45). Taken together, it appears that CLDN2-dependent regulation of paracellular Na^+^/H_2_O transport alone may not explain the complex effects of CLDN2 expression in IBD.

Notably, along with its role in paracellular transport, CLDN2 has been implicated in autophagy, cell proliferation, and migration in IECs as well as cancer stem cell regulation in CRC (18, 19, 47). In addition, CLDN2 is a target of EGFR, KGFR, vitamin D receptor, Wnt/β-catenin, pERK1/2, and pAKT signaling and immune cytokines, including IL-6, and IL-22, which play key role in MH (11, 17, 18, 43, 48–51). Many of these signaling pathways also promote neoplastic growth when deregulated, including in CRC (33, 52). Both, *Cldn2*KO mice and WT mice receiving EGFR inhibitor showed impaired recovery from colitis. Furthermore, in WT mice recovering from colitis, inhibiting EGFR signaling inhibited CLDN2 upregulation and MH. We have previously shown that EGFR activation is essential for colonic CLDN2 expression (16, 18). Our data that CLDN2 upregulation was localized primarily to the regenerating crypts and associated significantly with Ki67^+^ crypt cells aligned with a role for EGFR/CLDN2 signaling in regulating the crypt proliferation and/or migration. In this regard, our analysis of the coexpression of CLDN2 and Ki67 suggests that the recovery-associated CLDN2 upregulation was localized in crypt cells at the crypt base and the transit-amplifying zone. Further analysis using “single-cell analysis” will be needed for the true appreciation of specific crypt cells where CLDN2 expression is upregulated during MH, which is part of our ongoing studies. Taken together, it appears that inhibition of EGFR signaling inhibits CLDN2 expression to accelerate apoptosis of differentiated cells or reduced proliferation of the stem cell compartment, resulting in impaired recovery. Interestingly, pharmacological inhibition of EGFR signaling during CAC has context-dependent effects on CAC development (33, 53). This suggests a separation in CLDN2 versus EGFR functions in IBD and CAC, which deserves further investigation.

We have previously reported that CLDN2 overexpression in IECs promotes expression of antiapoptotic protein Bcl2 (10). In accordance with this, current data suggest a positive association between CLDN2 expression and IEC survival during colitis/recovery. Our results further show that CLDN2 promotes Survivin expression in promoting MH. As a member of the inhibitor of apoptosis family, Survivin is known for its antiapoptotic function and is highly expressed in regenerative tissue. We also found a positive association of *CLDN2* with a *BIRC5*-dependent transcriptional program. We validated the role of CLDN2 in modulating Survivin expression in multiple murine models of colitis and recovery and ex vivo studies using colonic crypts subjected to injury/repair. Of note, genetic inhibition of Survivin expression in the IECs in vivo promotes apoptosis and thus support our findings (40). A significant increase in CLDN2 expression along with Survivin in patients with IBD further supports our findings and the prognostic significance of the CLDN2/Survivin axis in IBD and CAC risk. EGFR signaling promotes Survivin expression (54). Our data that CLDN2 loss dysregulates the expression of proteins associated with the DDR further support a causal yet complex association of CLDN2 and Survivin in regulating IEC survival under stress. Moreover, expression of γH2A.X and P-21/Cip1 was upregulated in *Cldn2*KO mice, which is associated with genomic stability and protection from cancer development (40, 54). How CLDN2 regulates Survivin in IBD remains to be determined and is part of our ongoing studies. Overall, our data indicated that the EGFR/CLDN2/Survivin axis and DDR signaling play a crucial role in colitis/inflammation-associated MH.

Taken together, we describe what we believe to be a novel role for CLDN2 in protection from CAC, potentially by promoting MH. To our knowledge, this is the first report that describes the role of the dysregulated expression of a barrier-integral protein in promoting MH and, thus, barrier integrity as a means to limit disease severity and neoplastic growth. We believe that the outcome of this study will provide a better understanding of the role of CLDN2 in IBD and clinical management of patients, including CAC risk.

## Methods

### Cell lines and antibodies.

Caco-2 and HT-29 cells were purchased from ATCC. Cells were cultured in DMEM-high glucose and supplemented with 10% FBS and 100 U/mL penicillin-streptomycin. They were grown in a monolayer at 37°C with 5% CO_2_ and periodically tested for Mycoplasma contamination. Information about the antibodies used in this study is provided in Supplemental Table 2.

### Samples from patients with IBD.

All biopsy samples from patients with IBD were deidentified. Diagnosis of IBD was based on endoscopic findings and histological examination. In total, 21 biopsies with adjacent normal colon (14 from patients with ulcerative colitis and 7 from patients with Crohn’s disease with colon involvement) were taken from individuals who had chronic colitis and underwent endoscopy at University of Nebraska Medical Center (UNMC).

### IBD (GEO) transcriptomic data set analysis.

Publicly available whole-transcriptome data sets from patients with IBD (GSE48634, GSE53306, GSE38713, GSE16879, GSE36807, GSE9452, GSE13367, GSE14580, GSE75214, GSE59071, and GSE48958) (55–65) were downloaded from Gene Expression Omnibus (GEO; https://www.ncbi.nlm.nih.gov/geo/) using the GEOquery package in R programming language (66). All data were batch normalized and log_2_ transformed using the “sva” package in R programming language (67).

### Murine models of experimental colitis and CAC.

All mouse lines were on a C57BL/6 background. The Villin-*Cldn2*–transgenic (*Cldn2*TG) and *Cldn2*KO mice have been characterized previously (10, 44). Moreover, *Cldn2*TG and *Cldn2*KO mice are always bred with WT mice, and thus all the studies involving CLDN2-manipulated mice are done using littermate control mice. Age- and sex-matched C57BL/6, *Cldn2*TG, and *Cldn2*KO mice were used. The induction of acute and chronic DSS-induced colitis, colitis/recovery, and AOM/DSS-induced CAC was carried out as described previously (10, 11, 31, 68).

### Histological scoring.

H&E-stained colonic tissue sections were scored by pathologist in a masked manner using the following measures: inflammation (score of 0–3), percentage involved in inflammation (score of 1–4), depth of inflammation (score of 0–3), crypt damage (score of 1–4), and percentage involved in crypt damage (score of 1–4). The overall injury score was calculated using these individual scores, as explained in the Supplemental Table 3 (10).

### Determination of crypt regenerative index.

The extent of mucosal crypt regeneration was graded as described previously (69). Briefly, for epithelial regeneration, two pathologists graded the histological sections of control and experimental mouse colon in a masked fashion. A range from 0 to 3 was used to describe normal epithelium, epithelium not intact, and no tissue repair, respectively. The epithelial crypt regenerative index was determined by subtracting these scores from 3.

### Epithelial intrinsic models of intestinal epithelial injury.

Multiple models of intestinal epithelial injury and repair/regeneration were used, including DSS-, irradiation-, and 5FU-induced injuries (26, 70–72).

### Immunoblotting analysis.

Immunoblotting was performed using total lysates from the colons, crypts, and/or epithelial cells, as described previously (73).

### Mouse cytokine array analysis.

For cytokine analysis between *Cldn2*TG and WT mice, colonic tissues were homogenized in lysis buffer containing protease inhibitors and then centrifuged at 10,000*g* for 5 minutes to remove the cellular debris. Sample protein concentrations were estimated by Bradford Reagent (Bio-Rad) and normalized. Cytokine array was performed using the Proteome Profiler Mouse Cytokine Array Kit, Panel A (ARY006, R&D Systems) according to the manufacturer’s instruction. The relative expression of cytokines and chemokines was quantified using Image Lab software (Bio-Rad).

### Immunostaining analysis.

Immunofluorescence and/or immunohistochemical staining were done, as described previously (41). Imaging was done under identical microscope settings (for each antigen), and further processing was done using Adobe Photoshop.

### Staining intensity scoring.

Semiquantification of the intensity of immunofluorescence and immunohistochemical staining for CLDN2, Ki67, and Survivin was done using Nikon’s NIS-Elements-Basic software (Eclipse-*Ti* microscope, Nikon). The scoring of the CLDN2 immunostaining intensity in the epithelium of IBD biopsies was done by a pathologist. The intensity of CLDN2 expression was scored as follows: 0, negative; 1, weak; 2, moderate; and 3, strong. The criteria for grading the percentage scores were as follows: 0, 0% of cells were positive; 1, 1%–10% of cells were positive; 2, 11%–30% of cells were positive; 3, 31%–50% of cells were positive; 4, 51%–80% of cells were positive; and 5, >80% of cells were positive. The CLDN2 expression intensity was calculated by multiplying the percentage and intensity subscores.

### Colonoscopy-assisted intestinal wound healing assay.

A high-resolution Karl-Stroz mouse colonoscope was used. To study the mucosal wound healing process, colonoscope-assisted biopsies were performed, as described previously (74, 75). In brief, colonic wounds (2–3 wounds/mouse) were created using biopsy forceps in sedated mice under anesthetic (isoflurane inhalation). Wound healing process was longitudinally followed and digitally imaged using the colonoscope. Wound area of each biopsy region was calculated using Infinity Analyze software (Lumenera Corporation). The percentage of wound closure was quantified respective to day 0 of each genotype and then compared between *Cldn2*KO and WT mice. For immunofluorescence analysis, colons were opened longitudinally using a surgical microscope, and wounds were harvested from colons and frozen fixed in optimum cutting temperature formulation for further analysis.

### RNA isolation, qRT-PCR, and RNA-Seq analysis.

RNA isolation was done using the Quick-RNA Mini-Prep Kit (Zymo Research). RNA libraries were prepared, and sequencing was done using an Illumina NovaSeq6000 sequencer at the Genomics Core Facility, UNMC. The resulting sequencing data were quality tested and analyzed with established bioinformatics pipeline, which included mapping of the RNA-Seq to the reference genome using TopHat2, quantification of reads using “featureCounts” from the Subread package, differential analysis using DESeq2, and functional enrichment using FunSet and ConsensupathDB (76–79). For the RT-qPCR analysis, total RNA was reverse transcribed using the iScript cDNA Synthesis Kit (Bio-Rad). The PCR reactions were performed using antigen-specific primers (Supplemental Table 4). Each well contained SYBR Green Master Mix (Bio-Rad), respective primer set, and cDNA (30 ng). Samples were loaded in duplicates in 96-well plates and run on a Bio-Rad CFX connect (Bio-Rad) qPCR machine. Ct values were utilized to calculate fold change. Normalization was performed using β-actin amplification.

### Statistics.

All data presented are representative of at least 3 repeated experiments, unless stated otherwise. Mean values with SEM were used for independent 2-tailed Student’s *t* tests or 1-way ANOVA. Corrections for multiple comparisons were made using Tukey’s multiple comparison tests in Prism 9.3.1 (GraphPad Software Inc.). A *P* value of less than 0.05 was defined as statistically significant.

### Study approval.

Animal procedures were approved by the UNMC Institutional Animal Care and Use Committee (protocol 17.126.11FC). Deidentified biopsy specimens from patients with IBD who had undergone endoscopy procedures at UNMC were obtained and used in this study. The UNMC Institutional Review Board approved this study for clinical research.

### Data availability statement.

The RNA-Seq data were deposited in the GEO database (GSE207221). Values for all data points in graphs are reported in the Supporting Data Values file.

## Author contributions

RA, BK, RLT, and SKY conducted the experiments. RA, IT, and DKB performed the bioinformatics analyses. MKW and GAT performed pathological analyses. RA, PD, and ABS conceptualized the study and wrote manuscript. PD, ASY, and ABS critically reviewed the manuscript.

## Supplementary Material

Supplemental data

Supporting data values

## Version 1 10/10/2023

In-Press Preview

## Version 2 12/01/2023

Electronic publication
