# Supplementary material for: Claudin-2 protects against colitis-associated cancer by promoting colitis-associated mucosal healing
Source: J Clin Invest. 2023 Dec 1;133(23):e170771. doi: 10.1172/JCI170771 (PMC10688979; doi:10.1172/JCI170771)
Supplement: Supplemental data [file jci-133-170771-s034.pdf]

Claudin-2 protects from colitis-associated cancer by promoting colitis-associated mucosal healing

Rizwan Ahmad[1], Balawant Kumar[1], Ishwor Thapa[2], Raju Lama Tamang[1], Santosh K. Yadav[1], Mary K. Washington[3], Geoffrey A. Talmon[4], Alan S. Yu[5], Dhundy K. Bastola[2], Punita Dhawan[1,6,7] and Amar B. Singh[1,6,7]

[1]Department of Biochemistry and Molecular Biology, University of Nebraska Medical Center, Omaha, NE, [2]School of Interdisciplinary Informatics, University of Nebraska at Omaha, Omaha, NE, [3]Department of Pathology, Microbiology and Immunology, Vanderbilt University Medical Center, Nashville, TN, [4]Department of Pathology and Microbiology, University of Nebraska Medical Center, Omaha, NE, [5]Jared Grantham Kidney Institute, University of Kansas Medical Center, Kansas City, Kansas, [6]Fred and Pamela Buffett Cancer Center, University of Nebraska Medical Center, Omaha, NE and [7]VA Nebraska-Western Iowa Health Care System, Omaha, NE

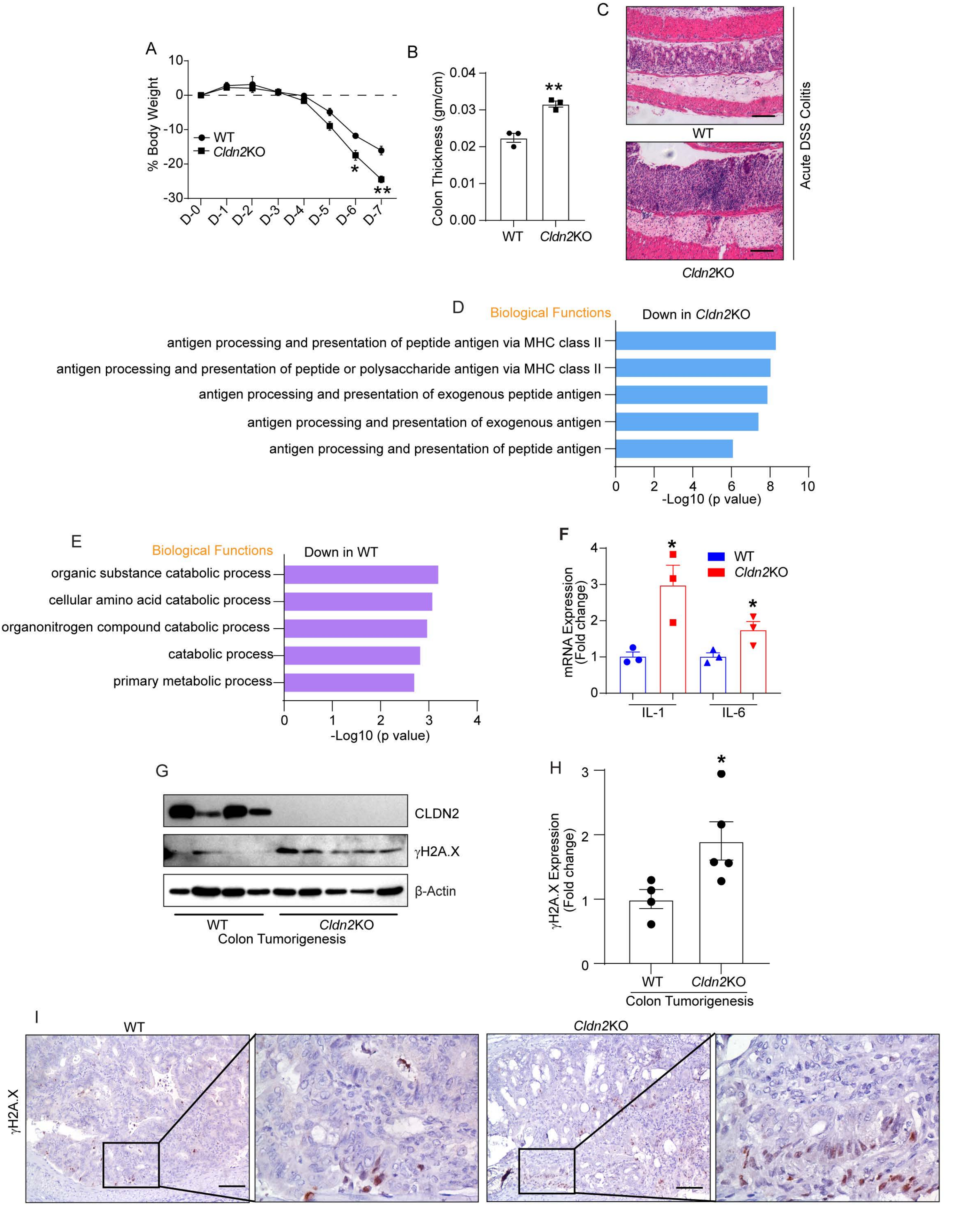

Supplementary Figure 1. *Cldn2*KO mice demonstrate severe disease when subjected to experimental colitis and CAC. *Cldn2*KO and WT mice were subjected to DSS-(Acute) colitis and CAC. (A) % weight decrease in *Cldn2*KO mice versus WT mice (n=3/group); (B) Colon Thickness (gm/cm; n=3/group); (C) Representative H&E images showing severe inflammation in *Cldn2*KO mice compared to WT mice; (D-E) GO biological function enrichment analysis based on the downregulated genes in *Cldn2*KO versus WT mice subjected to CAC (n=3/group); (F) Quantitative RT-qPCR analysis for inflammatory cytokines using colon of *Cldn2*KO and WT mice (n=3/group); (G-H) Immunoblots and densitometry analysis of DNA damage using γH2A.X antibody in *Cldn2*KO mice subjected to CAC (WT/*Cldn2*KO:n=4/5), and (I) IHC analysis of DNA damage using γH2A.X antibody in *Cldn2*KO mice subjected to CAC. Data in A, B, F, and H are presented as the mean ± SE; *P<0.05, and **P < 0.01 by 2-tailed unpaired t test. Images C and I: Scale bar=100 μM, Image J: Scale bar=200 μM. Scale bar=100 μM.

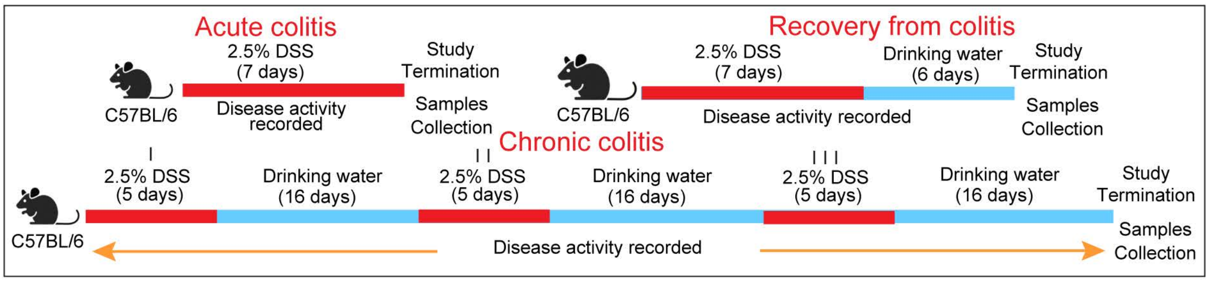

Supplementary Figure 2. Outline of the murine models of colitis used in the study.

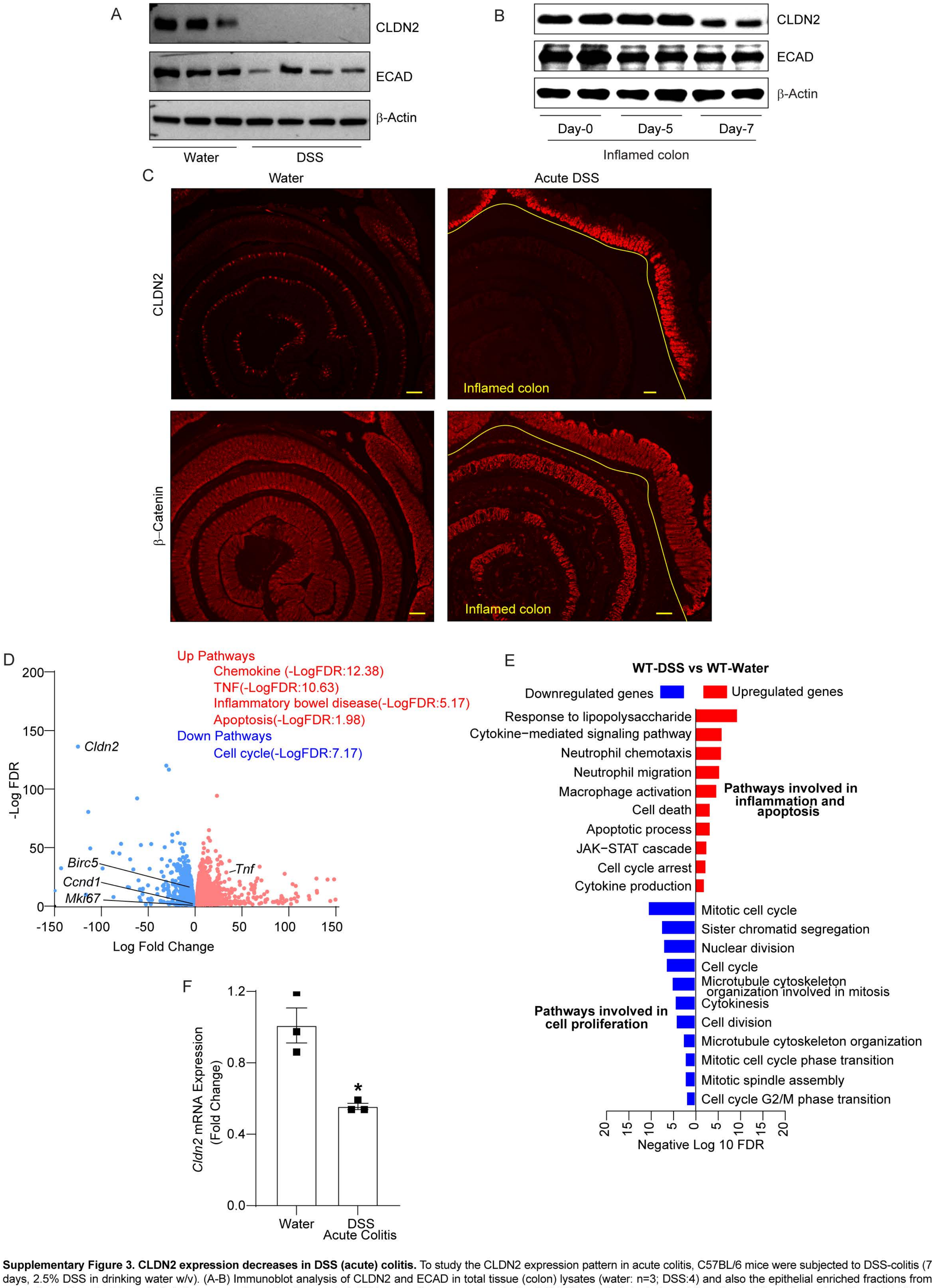

Supplementary Figure 3. CLDN2 expression decreases in DSS (acute) colitis. To study the CLDN2 expression pattern in acute colitis, C57BL/6 mice were subjected to DSS-colitis (7 days, 2.5% DSS in drinking water w/v). (A-B) Immunoblot analysis of CLDN2 and ECAD in total tissue (colon) lysates (water: n=3; DSS:4) and also the epithelial enriched fractions from respective colons (n=2/group). β-Actin was used as a loading control; (C) Immunofluorescence analysis of CLDN2 and β-Catenin in serial sections of the colon swiss-roles; (D) Volcano plot showing differentially expressed genes and KEGG pathways analysis during colitis using public databases (GSE98407 and GSE109728); (E) GO function analysis in C57BL6 mice subjected to DSS-colitis (n=3/group), and (F) RT-qPCR analysis for *Cldn2* in the colons of C57BL/6 mice (n=3/group) subjected to DSS-colitis. Data in F is presented as the mean ± SE; *P<0.05 by 2-tailed unpaired t test. Image C:Scale bar=200 μM.

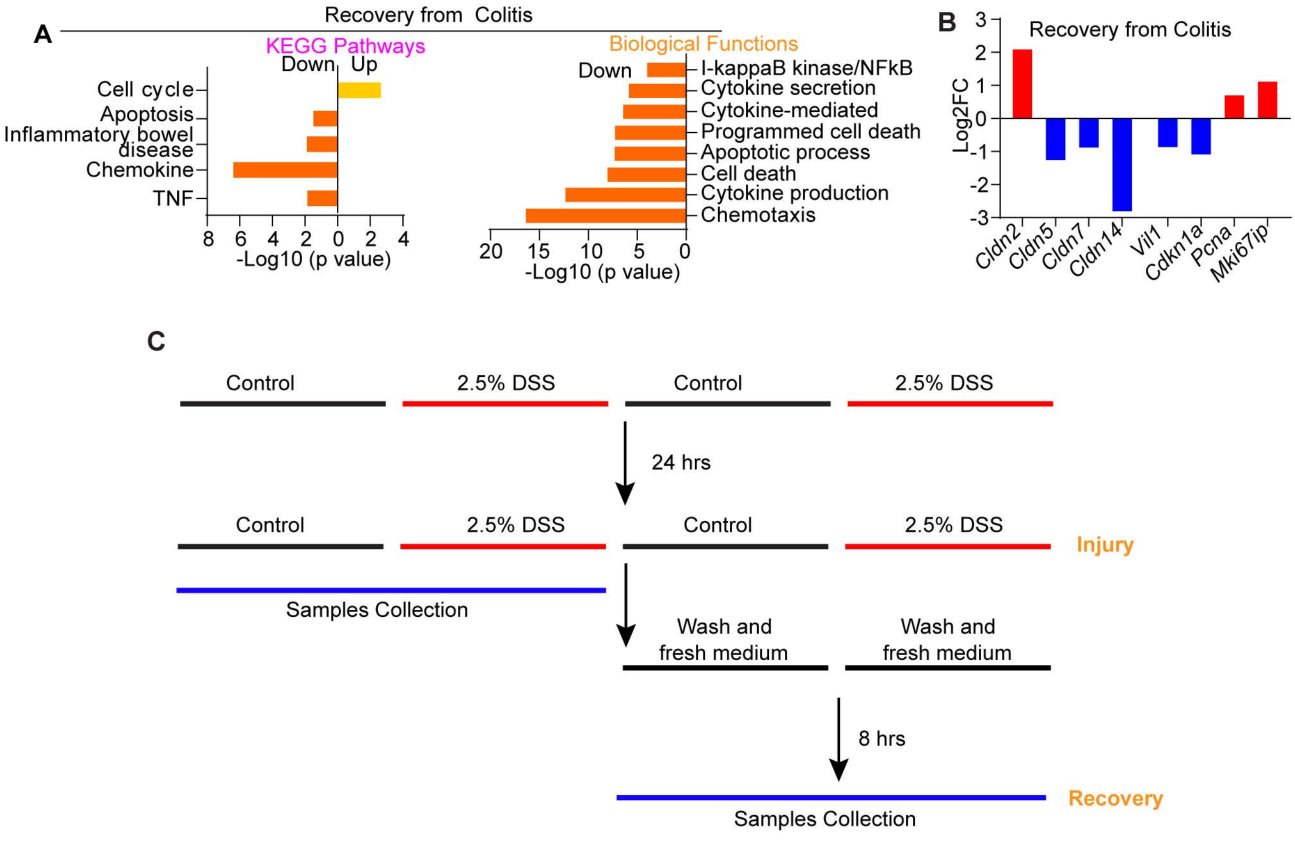

Supplementary Figure 4. (A) KEGG and GO enrichment analysis in mice recovering from DSS-(acute) colitis (n=3/group); (B) Differential gene regulation of claudin proteins and other proteins related with cell proliferation and differentiation during recovery from colitis (n=3/group) (C) Outline of the *in-vitro* model of DSS-induced intestinal epithelial cell Injury and recovery.

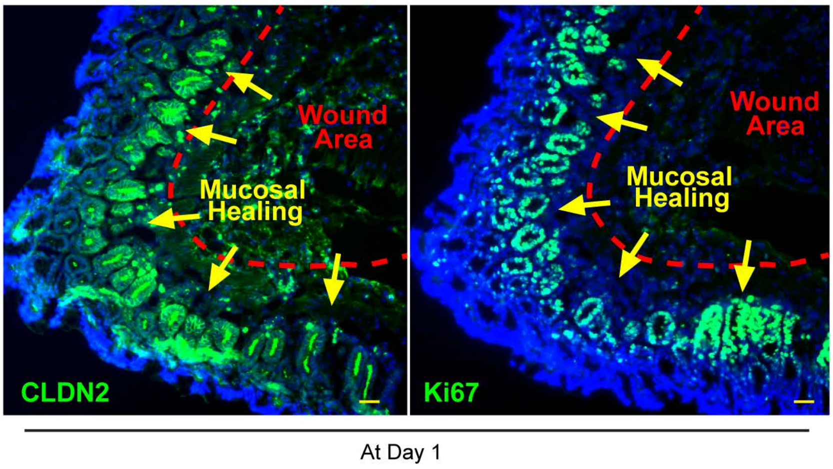

Supplementary Figure 5. Colonoscope-assisted mucosal wounding and healing in mice. Co-immunofluorescence staining of CLDN2 and Ki67 in the serial section of colonic biopsy associated epithelial injury/repair at day 1 (n=3).

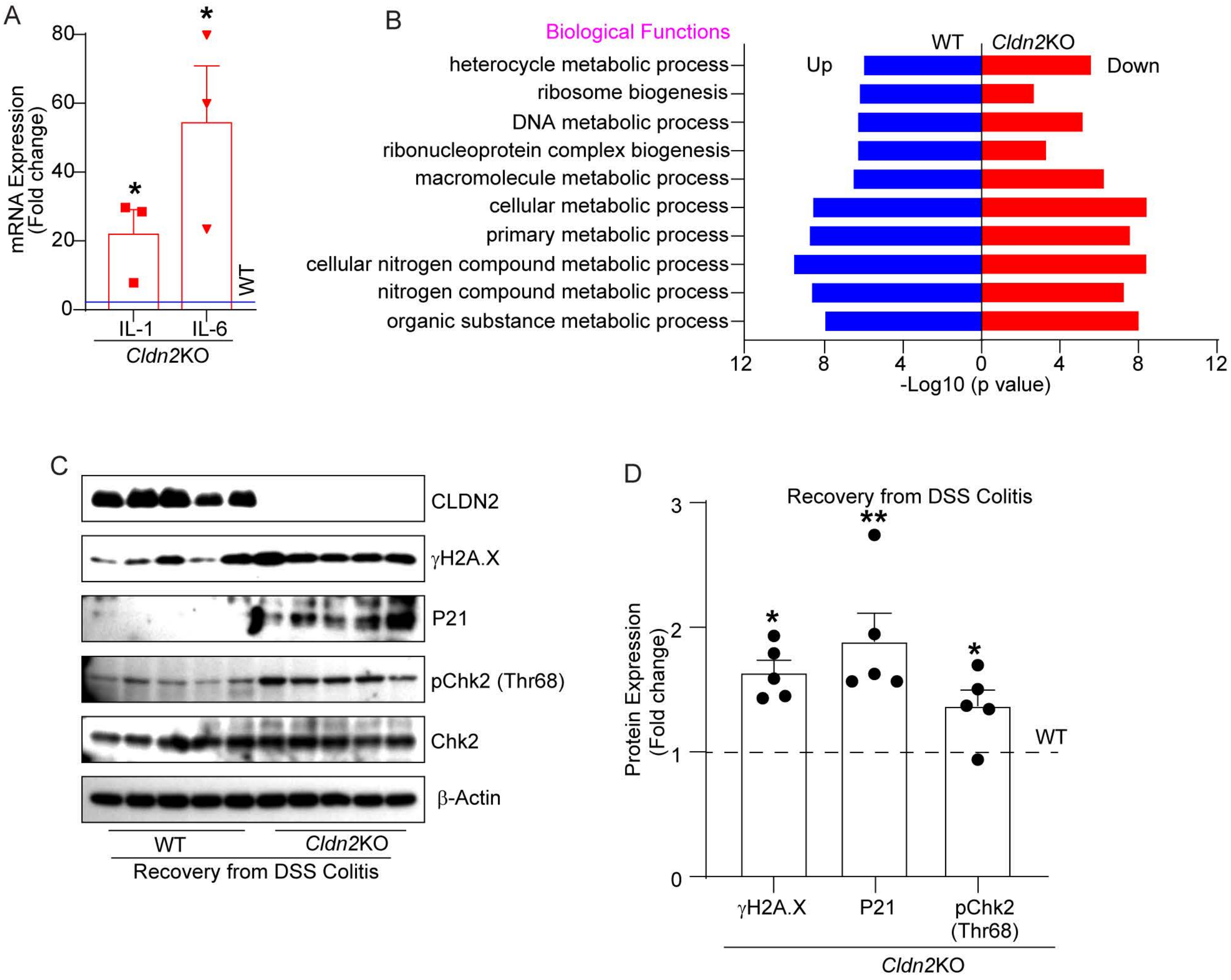

Supplementary Figure 6. *Cldn2*KO mice exhibit increased colonic inflammation and DNA damage during recovery from colitis. (A) Quantitative qRT-PCR analysis for Cytokines in colons from *Cldn2*KO (n=3) and WT mice (n=3), and (B) GO biological function analysis in *Cldn2*KO mice compared to WT (n=3/group); (C-D) Immunoblots and densitometry analysis of regulation of proteins involved in DNA damage response (DDR) pathway (n=5/group). Data in A and D are presented as the mean ± SE; *P<0.05, and **P < 0.01 by 2-tailed unpaired t test.

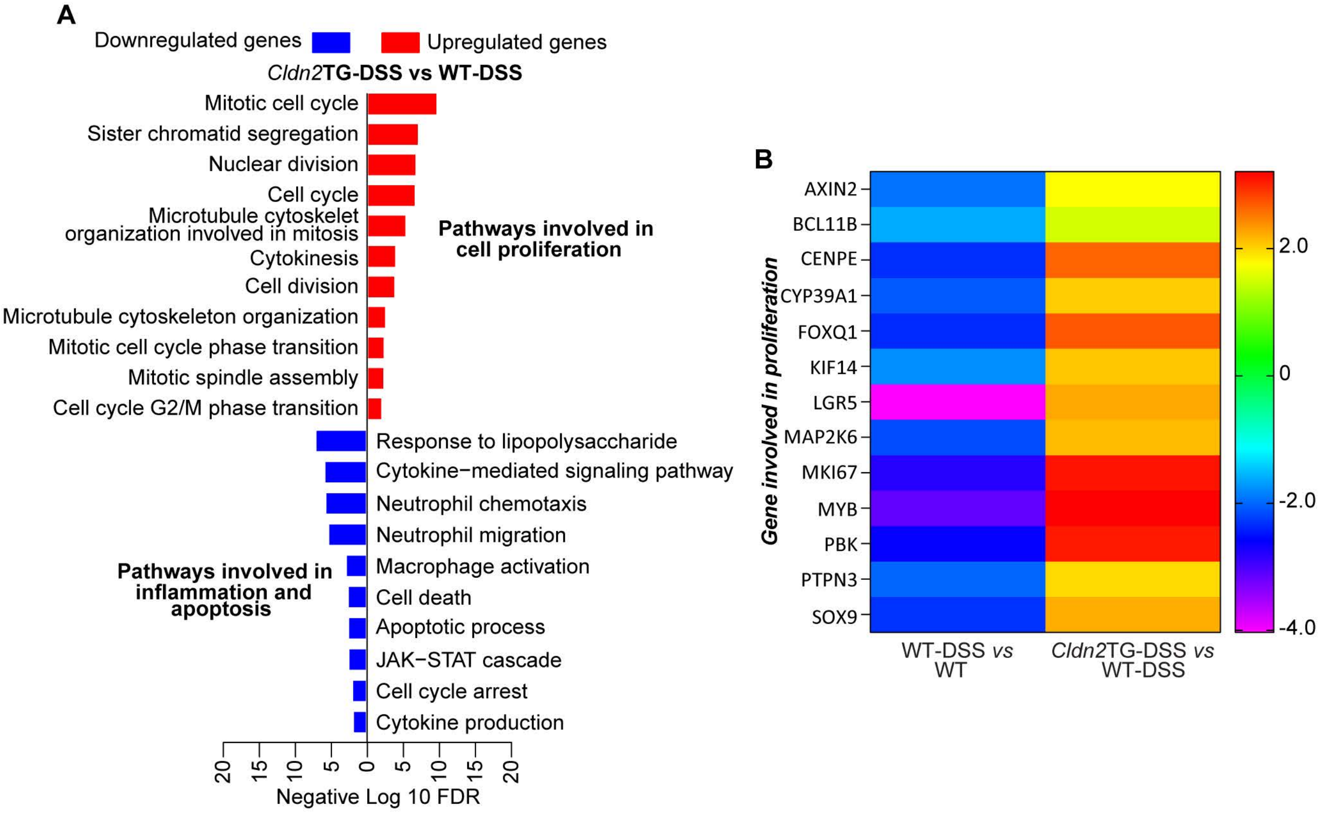

Supplementary Figure 7. Analysis of the high throughput transcriptome data (microarray; DEGs) in Villin-*Cldn2*TG mice subjected to DSS-colitis. (A) Signaling pathways associated with proliferation and inflammation in Villin-*Cldn2*TG and WT mice, and (B) Proliferative genes are upregulated in Villin-*Cldn2*TG mice versus WT mice (n=3/group).

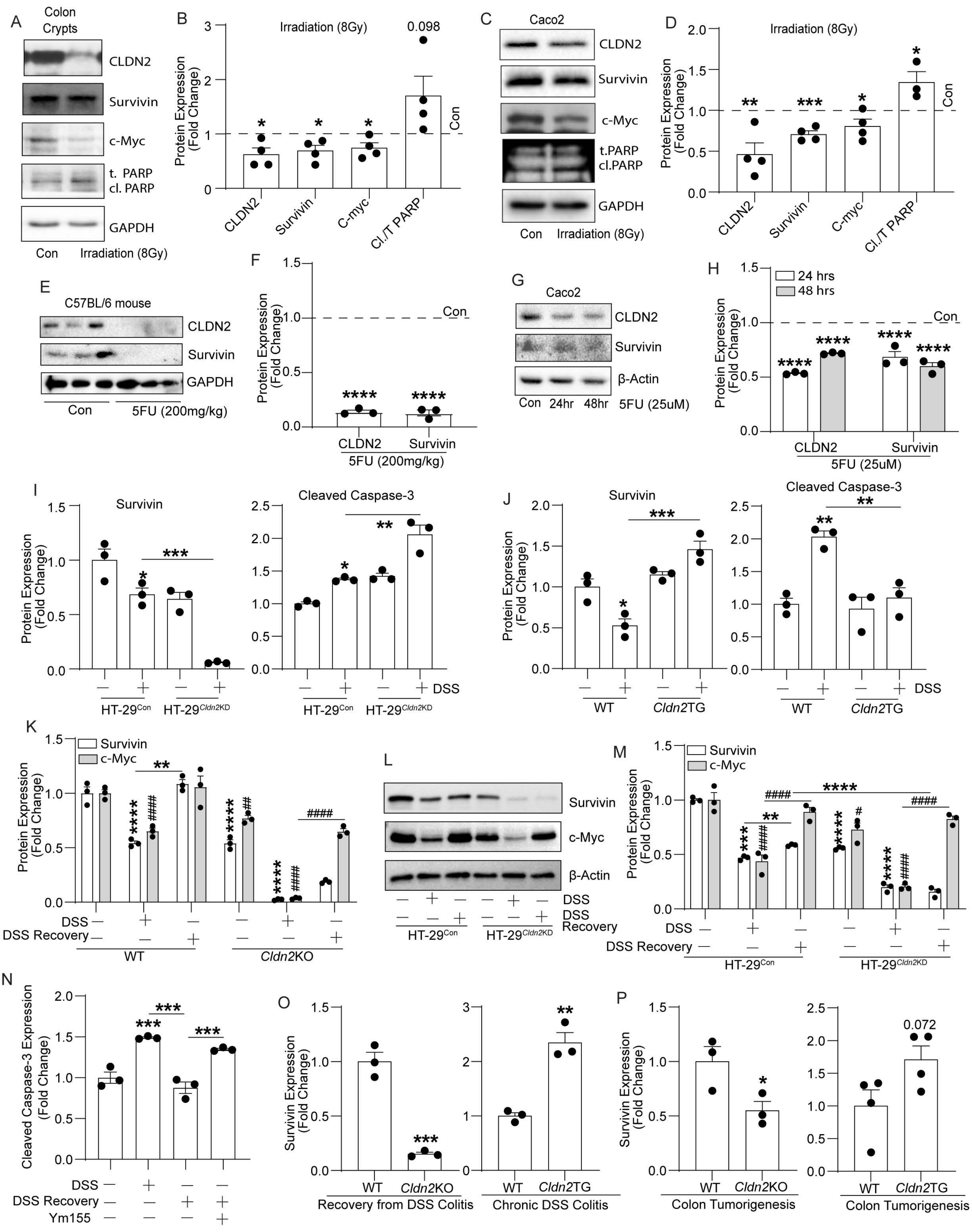

Supplementary Figure 8. CLDN2 expression helps promote colitis-associated epithelial repair/regeneration by regulating Survivin. (A-D) Immunoblots and densitometric analysis for CLDN2, Survivin, cleaved PARP and c-Myc in colon crypts and cells subjected to irradiation injury (n=4 independent experiments); (E-H) Immunoblot densitometric analysis using anti-CLDN2 and -Survivin antibody in mice (n=3/group) and colonic epithelial cells subjected to 5FU treatment (n=3 independent experiments); (I-J) Densitometry analysis of immunoblots using lysates from the DSS-treated colonic epithelial cells and Ex-vivo 3D-culture of the colon crypts from Control and *Cldn2*KD cells and WT and Villin-*Cldn2*TG mice (n=3 independent experiments); (K) Immunoblots and densitometric analysis in isolated colonic cells from *Cldn2*KO and WT mice subjected to the DSS-induced injury/repair (n=3 independent experiments); (L-M) Immunoblots and densitometric analysis in HT-29^{Cldn2KD} cells subjected to the DSS-induced injury/repair (n=3 independent experiments); (N) Densitometry analysis of immunoblots from Caco-2 cells subjected to DSS-induced injury/repair with or without inhibitors of the Survivin (n=3 independent experiments); and (O-P) Densitometry analysis of Survivin in *Cldn2*KO (n=3), Villin-*Cldn2*TG (n=3) versus WT (n=3) mice subjected to recovery from colitis, chronic colitis, and CAC tumorigenesis. Data in B, D, F, H, O, and P are presented as the mean ± SE; *P<0.05, **P<0.01, ***P<0.001 and ****P<0.0001 by 2-tailed unpaired t test. Data in I,-K, M, and N are presented as the mean ± SE; *,#P<0.05, **,##P<0.01, ***P<0.001 and ****,####P<0.0001 by 1-way ANOVA with Tukey's test.

Supplementary Table 1: GO biological processes associated with mucosal healing among IBD patients and *Cldn2*KO mice versus WT: List of overlap genes.

Genes Symbol (Human)	Genes Symbol (mouse)	IBD patient's vs Normal (Log2FC)	WT DSS Recovery vs WT naïve (Log2FC)	*Cldn2*KO DSS Recovery vs WT DSS Recovery (Log2FC)
AGR2	*Agr2*	93.89879	2.177104	-1.27248
BET1	*Bet1*	14.49927	1.169584	-0.73876
CENPK	*Cenpk*	13.35544	0.890634	-1.24903
CLDN2	*Cldn2*	16.95404	2.129701	-9.93198
DSP	*Dsp*	-48.0657	-1.22025	1.122645
DST	*Dst*	-4.66525	-0.87268	1.169607
DYRK2	*Dyrk2*	-21.1406	-1.07438	1.098311
ELF4	*Elf4*	-9.04182	-1.04502	0.925597
EPS8L3	*Eps8l3*	-15.1389	-1.28204	1.474975
EXOSC7	*Exosc7*	7.058332	0.720652	-0.72429
FNIP2	*Fnip2*	-9.91554	-1.15263	1.306066
IKBIP	*Ikbip*	15.31687	0.937849	-0.9175
JUP	*Jup*	-17.9472	-0.93463	0.964127
KLF4	*Klf4*	-31.3081	-1.36817	0.801889
MARK2	*Mark2*	-5.71611	-0.76724	0.760024
MICALCL	*Micalcl*	-7.54921	-0.70616	0.795552
NHLRC2	*Nhlrc2*	-2.83041	-0.93788	0.963795
NOP10	*Nop10*	18.7255	1.295753	-0.89378
OSTC	*Ostc*	45.67739	0.982062	-0.88792
PHACTR4	*Phactr4*	-11.2507	-0.78475	0.819607
PYCR1	*Pycr1*	11.44125	2.086356	-1.16935
SIPA1L3	*Sipa1l3*	-12.6488	-1.09369	1.090884
SRGAP1	*Srgap1*	-5.43438	-1.45302	1.142119
TPD52L1	*Tpd52l1*	12.99194	4.540992	-1.01363

Supplementary Table 2 : List of antibodies

S.N.	Antibody	Company	Catalog Number
1	Claudin 2 Mouse monoclonal antibody (12H12)	Invitrogen	32-5600
2	E-cadherin Mouse monoclonal antibody	BD biosciences	610181
3	p27 Antibody (F-8)	Santa Cruz Biotechnology	sc-1641
4	P21 polyclonal antibody	Proteintech	10355-1-AP
5	Cleaved Caspase 3 antibody	Cell signaling Technology	9661S
6	Cleaved PARP antibody	Cell signaling Technology	5625S
7	Phospho-Histone H2A.X (Ser139) (20E3)	Cell signaling Technology	9718
8	Phospho-Chk2 (Thr68) (C13C1) Rabbit mAb	Cell signaling Technology	2197
9	Chk2 (D9C6) Rabbit mAb	Cell signaling Technology	6334
10	Bcl-2	BD Transduction Laboratories™	610538
11	Phospho-Stat3 (Tyr705)	Cell signaling Technology	9131
12	Stat3 (124H6) Mouse mAb	Cell signaling Technology	9139
13	Phospho-NF-κB p65 (Ser536) (93H1) Rabbit mAb	Cell signaling Technology	3033
14	NF-κB p65 (D14E12) XP® Rabbit mAb	Cell signaling Technology	8242
15	c-Myc	Santa Cruz Biotechnology	sc-40
16	Survivin (71G4B7) Rabbit mAb	Cell signaling Technology	2808
17	Mouse Sca-1/Ly6 Antibody	R&D Systems	MAB1226
18	Ki67 (Rabbit monoclonal antibody)	Cell signaling Technology	12202s
19	pERK (Rabbit monoclonal antibody)	Cell signaling Technology	4370S
20	ERK (Rabbit monoclonal antibody)	Cell signaling Technology	9102S
21	GAPDH	Proteintech	60004-1-Ig

Supplementary Table 3: Injury Scoring criteria.

**Inflammation (A)**<br><br>0-3	**% involved by inflammation (B)**<br><br>1-4	**Depth of inflammation (C)**<br><br>0-3	**Crypt damage (D)**<br><br>1-4	**% involved by crypt damage (E)**<br><br>1-4	**Injury score calculation**
0(None)<br>1(Mild)<br>2(Moderate)<br>3(Severe)	1 (1 to 25%)<br>2 (26 to 50%)<br>3 (51 to 75%)<br>4 (>75%)	0(None)<br>1(Mucosa)<br>2(Submucosa)<br>3(Transmural)	1(Basal 1/3 damaged)<br>2(Basal 2/3 damaged)<br>3(Only surface intact)<br>4(Entire crypt and surface lost)	1 (1 to 25%)<br>2 (26 to 50%)<br>3 (51 to 75%)<br>4 (>75%)	=(A+C)xB+(DxE)

Supplementary Table 4: List of real-time qPCR primers sets.

Gene	Sense Primer	Antisense Primer
Mouse		
Actin	5'-CCAGAGCAAGAGAGGTATCC-3'	5'-CTGTGGTGGTGAAGCTGTAG-3'
IL-6	5'-AGAGACTTCCATCCAGTTGC-3'	5'-TCCTTAGCCACTCCTTCTGT-3'
IL-1	5'-CTCCATGAGCTTTGTACAAGG-3'	5'- TGCTGATGTACCAGTTGGGG-3'
Cldn2	5'-GATTGGAGAGGCTCTGTACTTG-3'	5'-TAGTTGGTACGATTGCCCTG-3'
Human		
GAPDH	5' GGCAAATTCAACGGCACAGT-3'	5' AGATGGTGATGGGCTTCCC-3'
Ki67	5'- TGACCCTGATGAGAAAGCTCAA-3'	5'- CCCTGAGCAACACTGTCTTTT-3'
P21	5'- TGGAGACTCTCAGGGTCGAAA -3'	5'- GGCGTTTGGAGTGGTAGAAATC-3'
